# Cholestane-3β, 5α, 6β-triol Suppresses Proliferation, Migration, and Invasion of Human Prostate Cancer Cells

**DOI:** 10.1371/journal.pone.0065734

**Published:** 2013-06-13

**Authors:** Ching-Yu Lin, Chieh Huo, Li-Kuo Kuo, Richard A. Hiipakka, Richard Baker Jones, Hui-Ping Lin, Yuwen Hung, Liang-Cheng Su, Jen-Chih Tseng, Ying-Yu Kuo, Yu-Ling Wang, Yasuhisa Fukui, Yung-Hsi Kao, John M. Kokontis, Chien-Chih Yeh, Linyi Chen, Shiaw-Der Yang, Hsiao-Hui Fu, Ya-Wen Chen, Kelvin K. C. Tsai, Jang-Yang Chang, Chih-Pin Chuu

**Affiliations:** 1 Institute of Cellular and System Medicine, National Health Research Institutes, Miaoli, Taiwan; 2 Translational Center for Glandular Malignancies, National Health Research Institutes, Miaoli, Taiwan; 3 National Institute of Cancer Research, National Health Research Institutes, Miaoli, Taiwan; 4 Department of Life Sciences, National Central University, Taoyuan, Taiwan; 5 Division of Pulmonary and Critical Care Medicine, Department of Internal Medicine, Mackay Memorial Hospital, Taipei, Taiwan; 6 Ben May Department for Cancer Research, The University of Chicago, Illinois, United States of America; 7 Institute for Genomics and Systems Biology, The University of Chicago, Illinois, United States of America; 8 Institute of Molecular and Cellular Biology, National Tsing Hua University, Hsinchu, Taiwan; 9 Institute of Molecular Medicine, National Tsing Hua University, Hsinchu, Taiwan; 10 Division of Colon and Rectal Surgery, Taoyuan Armed Forces General Hospital, Taoyuan, Taiwan; 11 Department of Surgery, Taoyuan Armed Forces General Hospital, Taoyuan, Taiwan; 12 Graduate Program for Aging, China Medical University, Taichung, Taiwan; 13 Ph.D. Program in Tissue Engineering and Regenerative Medicine, National Chung Hsing University, Taichung, Taiwan; Innsbruck Medical University, Austria

## Abstract

Oxysterols are oxidation products of cholesterol. Cholestane-3β, 5α, 6β-triol (abbreviated as triol) is one of the most abundant and active oxysterols. Here, we report that triol exhibits anti-cancer activity against human prostate cancer cells. Treatment of cells with triol dose-dependently suppressed proliferation of LNCaP CDXR-3, DU-145, and PC-3 human prostate cancer cells and reduced colony formation in soft agar. Oral administration of triol at 20 mg/kg daily for three weeks significantly retarded the growth of PC-3 xenografts in nude mice. Flow cytometric analysis revealed that triol treatment at 10–40 µM caused G1 cell cycle arrest while the TUNEL assay indicated that triol treatment at 20–40 µM induced apoptosis in all three cell lines. Micro-Western Arrays and traditional Western blotting methods indicated that triol treatment resulted in reduced expression of Akt1, phospho-Akt Ser473, phospho-Akt Thr308, PDK1, c-Myc, and Skp2 protein levels as well as accumulation of the cell cycle inhibitor p27^Kip^. Triol treatment also resulted in reduced Akt1 protein expression in PC-3 xenografts. Overexpression of Skp2 in PC-3 cells partially rescued the growth inhibition caused by triol. Triol treatment suppressed migration and invasion of DU-145, PC-3, and CDXR-3 cells. The expression levels of proteins associated with epithelial-mesenchymal transition as well as focal adhesion kinase were affected by triol treatment in these cells. Triol treatment caused increased expression of E-cadherin protein levels but decreased expression of N-cadherin, vimentin, Slug, FAK, phospho-FAK Ser722, and phospho-FAK Tyr861 protein levels. Confocal laser microscopy revealed redistribution of β-actin and α-tubulin at the periphery of the CDXR-3 and DU-145 cells. Our observations suggest that triol may represent a promising therapeutic agent for advanced metastatic prostate cancer.

## Introduction

Prostate cancer is the second most frequently diagnosed cancer of men and the fifth most common cancer overall in the world. In 2008, more than 899,000 new cases were diagnosed (GLOBOCAN 2008 database, version 1.2). In Western countries, prostate cancer is the most common non-cutaneous carcinoma of men. According to the statistics of Surveillance Epidemiology and End Results (SEER) of the National Cancer Institute, more than 240,000 men were diagnosed with and more than 28,000 men died of prostate cancer in 2012 in the United States. Although surgery is often successful for organ-confined prostate cancer, androgen ablation therapy is the primary treatment for metastatic prostate cancer. Unfortunately, most prostate cancer patients receiving androgen ablation therapy will ultimately develop recurrent, castration-resistant tumors within 1–3 years after treatment. The median overall survival time is 1–2 years after cancer relapse [1], [2]. No effective standard therapy exists for patients that relapse with advanced prostate cancer. Chemotherapy is often used to treat metastatic hormone-refractory prostate cancer 2,3. However, chemotherapies generally show little effect on prolonging survival. Therefore, new treatments for advanced prostate cancers are needed.

Oxysterols are oxidation products of cholesterol. Oxysterols play essential roles in regulating cholesterol homeostasis, platelet aggregation, apoptosis, and protein prenylation [4]. However, oxysterols are associated with development of atherosclerosis, neurological disease, and cancers [4]. Certain oxysterols have been reported to exhibit anticancer effects, possibly via modulation of cholesterol efflux, Akt, or liver X receptors (LXRs) [5], [6]. For example, treatment with 22(R)-hydroxycholesterol, 24(S)-hydroxycholesterol, 7α-hydroxycholesterol, 7β-hydroxycholesterol, 25-hydroxycholesterol, and 5α,6α-epoxycholesterol suppressed the proliferation of human prostate, breast, colon, lung, and leukemia cancer cells [7]–[14]. These oxysterols caused either G1 cell cycle arrest [7]–[11] or apoptosis in cancer cells [12]–[14]. Therefore, oxysterols with cytotoxic activity might be a potential therapeutic agent for advanced prostate cancers.

Cholestane-3β, 5α, 6β-triol (abbreviated as triol) is one of the most abundant oxysterols. Triol is derived from cholesterol by oxidation via formation of 5α, 6α-epoxycholesterol and 5β, 6β-epoxycholesterol [15], [16] as intermediates. Previously, 5α, 6α-epoxycholesterol was reported to exhibit anti-cancer activity [13]. In this study, we examined the ability of triol to suppress the proliferation of advanced human prostate cancer cell lines both *in vitro* and *in vivo*. We applied Micro-Western Arrays, a recently developed antibody-based, high-throughput Western blotting assay [17]–[20], to study the signaling proteins affected by triol in advanced prostate cancer cells. Our observations suggested that triol may represent a promising therapeutic agent for advanced metastatic prostate cancer.

## Materials and Methods

### Materials

Cholestane-3β, 5α, 6β-triol was purchased from Sigma (St. Louis, MO, USA). Matrigel was purchased from BD Bioscience (San Jose, CA, USA).

### Cell Culture

Prostate cancer cell lines PC-3, DU-145, and LNCaP sublines were a gift from the laboratory of Professor Shutsung Liao (The University of Chicago, IL, USA). These cell lines were previously described [7], [8], [10], [21]–[27]. LNCaP CDXR-3 cells were passaged and maintained as described previously [7], [8], [10], [11], [21]–[23], [27]. DU-145 and PC-3 cells were maintained in DMEM (Gibco/Invitrogen, Carlsbad, CA, USA) supplemented with 10% fetal bovine serum (FBS; Atlas Biologicals, Fort Collins, CO, USA), penicillin (100 U/ml), and streptomycin (100 µg/ml) (complete medium) as previously described [28]. The PZ-HPV-7 cells were purchased from Bioresource Collection and Research Center (BCRC, Hsinchu City, Taiwan) and were cultured in a keratinocyte-serum free medium (Gibco/Invitrogen) with 5 ng/ml human recombinant epidermal growth factor and 50 µg/ml bovine pituitary extract.

### Cell Proliferation Assay

Cells were seeded at a density of 3×10^3^ cells/well in 100 µl complete medium in 96-well plates. Proliferation assays were performed under maintenance conditions (DMEM with 10% FBS for PC-3 and DU-145; DMEM with 10% CS-FBS for LNCaP CDXR-3). Relative cell number was analyzed by measuring the DNA content of cell lysates with the fluorescent dye Hoechst 33258 (Sigma, St. Louis, MO, USA) as described previously [8], [10], [11], [21], [23], [24], [27], [28]. All readouts were normalized to the average of the control condition (no triol treatment) in each individual experiment. The experiment was repeated three times. Ten wells were used for each condition. The mean and standard deviation represented the average and standard deviation respectively of the results from all 30 wells in the three experiments.

### Cell Viability Assay

Cells were seeded at a density of 3×10^3^ cells per well in a 96-well plate (BD Bioscience). After 24 hrs, the cells were treated with increasing concentrations of triol for 48 hrs or 96 hrs. Cell viability was assessed by an MTT (3,4,5-dimethylthiazol-2-yl)-2-5-diphenyltetrazolium bromide) assay [29]. The amount of formazan was determined by measuring the absorbance at 560 nm using an Tecan GENios™ plate reader (Tecan group Ltd, Männedorf, Switzerland) [29]. All results were normalized to the average of the control condition (no triol treatment) in each individual experiment. The experiment was repeated three times. Each time ten wells were utilized for each condition. The mean and standard deviation represented the results from all 30 wells in the three experiments.

### Flow Cytometric Analysis

Cells were seeded at a density of 5×10^5^ cells in 10-cm dishes in 10 mL of media and cultured for 24 hrs before addition of triol. After 48 hrs of culture in the presence of various concentrations of triol, cells were removed with trypsin and fixed in 70% ethanol in phosphate buffered saline (PBS) overnight at −20°C. Fixed cells were washed with PBS, treated with 0.1 mg/mL RNase A in PBS for 30 min, and then suspended in PBS containing 50 µg/mL propidium iodide. Cell cycle profiles and distributions were determined by flow cytometric analysis of cells using a BD Facscan flow cytometer (BD Biosciences). The cell cycle distribution was analyzed using ModFit LT software (Verity Software House, Topsham, ME, USA) as described [10], [23], [24], [26], [27].

### Soft Agar Colony Formation Assay

PC-3 and LNCaP CDXR-3 cells (8×10^3^) were suspended in 0.3% low melting agarose (Lonza, Allendale, NJ, USA) containing DMEM with 10% FBS or 10% charcoal-stripped FBS (CS-FBS), respectively, and then layered on top of 3 ml of solidified 0.5% low melting agarose containing DMEM medium with 10% FBS (PC-3) or 10% CS-FBS (CDXR-3) in 6 cm dishes. Cells were allowed to grow at 37°C with 5% CO2 for 14 days (PC-3) or 17 days (CDXR-3). The plates were stained with 0.005% crystal violet in 30% ethanol for 6 hrs to detect cell colonies.

### TUNEL Assay

Cells were cultured on cover slips in 24 wells and were treated with 0, 20, and 40 µM triol for 48 or 96 hrs. Cells were rinsed twice with PBS and subjected to the TUNEL assay using ApoAlert DNA Fragmentation Assay Kit (catalog no. 630108 from Clontech, Mountain View, CA, USA) according to the manufacture's instruction. The TUNEL-stained cells were observed with Olympus confocal microscope at 200 × (FV300, Olympus, Tokyo, Japan).

### Ethics Statement

This study was carried out in strict accordance with the recommendations in the Guide for the Care and Use of Laboratory Animals of the National Institutes of Health (Taiwan). The protocol was approved by the National Health Research Institutes Institutional Animal Care and Use Committee (NHRI-IACUC-101046-A). Mice were kept under controlled environmental conditions (22°C, 12 hrs alternate light-dark cycles, 50% humidity, food and water ad libitum). Anesthesia (Isoflurane 1%) was given to reduce pain in mice during inoculation of prostate cancer cells. Animal care was conducted in accordance with the standard ethical guidelines (National Institutes of Health's “Guide for the care and use of Laboratory animals” and Clinical laboratory animal medicine (K. Hrapkiewicz, L. Medina, and D.D. Holmes, 1998, Iowa State University Press). The experiment was designed to minimize the number of nude mice used.

### Tumor Xenografts in Athymic Mice

Experiments involving mice were approved by National Health Research Institutes Institutional Animal Care and Use Committee (NHRI-IACUC-101046-A). Male Balb/c nu/nu mice (NCI Frederick, MD), 6–8 weeks of age, were injected subcutaneously in both flanks with 5×10^5^ PC-3 cells suspended in 0.5 ml of Matrigel (BD Bioscience, Franklin Lakes, NJ, USA). Monitoring of tumor growth was started one week after tumor inoculation. Mice were separated into control and treatment groups. 5 mice carrying 10 tumors comprised the control group and 5 mice carrying 8 tumors comprised the treatment group. Three weeks after inoculation of PC-3 cells, mice in the treatment group were treated orally 5 days/week with 20 mg/kg triol. Triol was administered in a vehicle containing 1% Tween 20, 25% dimethylsulfoxide in PBS. Mice in the control group were gavaged with vehicle only. Triol treatment was started 21 days after inoculation of cells and continued for 21 days. Tumors were measured weekly using calipers and volume was calculated using the formula volume = length×width×height×0.52 [7], [21]–[23].

### Luciferase-reporter Assay

PC-3 cells were seeded at 2×10^5^ cells/well in a 12-well plate in DMEM containing 10% FBS. 24 hrs after plating, PC-3 cells were transfected with pRL-TK-Renilla luciferase plasmid (normalization vector; 5 ng/well), pSG5RXRα and pSG5LXRα (400 ng/well ), 4xDR4Δ56cfos pGL3 (reporter gene vector; 500 ng/well) using the PolyJet™ in vitro DNA transfection reagent (SigmaGen Laboratories, Rockville, MD, USA). 24 hrs after transfection, cells were treated with increasing concentrations of triol or T0901317. After an additional 24 hrs, cells were lysed in 100 µl passive lysis buffer (Promega, Madison, WI, USA) and luciferase activity was measured using a Dual-Luciferase kit (Promega) in a 20/20^n^ luminometer Turner Biosystems.

### Western Blotting Analysis

Cells samples were lysed in SDS lysis buffer (240 mM Tris-acetate, 1% SDS, 1% glycerol, 5 mM EDTA pH 8.0, 0.1 mM dithiothreitol) containing the protease inhibitors 1 mM 4-(2-aminoethyl)benzenesulfonyl fluoride (AEBSF), 0.8 µM aprotinin, 40 µM bestatin, 14 µM E-64, 20 µM leupeptin, 15 µM Pepstatin A (Sigma-Aldrich, P8340) and the phosphatase inhibitors cantharidin, bromotetramisole and microcystin LR (Sigma-Aldrich, P2850). Tissues samples were prepared from tissue homogenized in 2× Laemmli buffer as previously described [7], [21]–[24]. Expression of proteins including Akt1, Skp2, phospho-Akt Ser473, phospho-Akt Thr308, PDK1, phospho-p44/42 MAPK Thr202/Tyr204, CDK2, CDK6, fatty acid synthase (FASN), GSK-3α, GSK-3β, Rb, cyclin B1, cyclin D1, phospho-p38 MAPK Thr180/Tyr182, phospho-PDK1 Ser241, phospho-Rb Ser780, FAK, and MMP9 was detected using antibodies from Cell Signaling Technology (Danvers, MA, USA). E-cadherin, N-cadherin, and p27^Kip1^ antibodies were from BD BioSciences (San Jose, CA, USA). Antibodies for detection of c-Myc, caspase 3, caspase 8, caspase 9, PTEN were from Epitomics (Burlingame, CA, USA). Slug antibody was purchased from Abgent (San Diego, CA, USA). Vimentin antibody was from Thermo (Waltham, Massachusetts, USA). The antibodies against Akt1, Akt2, Akt3, Bcl-2, P53, Cdk4, NF-κB, cyclin A, cyclin E1, cyclin E2, E2F-1, phospho-GSK-3α Ser21, phospho-GSK-3β Ser9, phospho-c-Myc Thr58/Ser62, phospho-PTEN Ser385, phospho-FAK Ser722, phospho-FAK Tyr861, and α-tubulin were from Millipore (Billerica, MA, USA). Antibodies detecting α-tubulin, β-actin, and GAPDH were purchased from Novus (Littleton, CO, USA). Antibodies for Skp2, p21^Cip^, and p27^Kip^ were from Santa Cruz (Dallas, TX, USA). Horseradish peroxidase-conjugated anti-rabbit and anti-mouse IgG secondary antibodies were from Santa Cruz. The signal of horseradish peroxidase labeled secondary antibodies was detected by enhanced chemoluminescence reaction (ECL Western Blotting detection kit) (PerkinElmer, Waltham, MA, USA). GAPDH, α-tubulin, and β-actin were used as loading controls.

### Micro-Western Arrays

CDXR-3 and DU-145 cells were treated with vehicle (DMSO) or 10 µM triol for 48 hrs. Micro-Western Arrays were performed to measure protein expression and modification as previously described [17], [18], [20], [24].

### Skp2 Overexpression in PC-3 Cells

Ectopic expression of Skp2 was achieved by infecting PC-3 cells with a retrovirus generated from the LPCX plasmid containing wild-type human Skp2 cDNA as described [10]. Puromycin-resistant colonies were expanded and screened for increased Skp2 protein expression by Western blot analysis. PC-3 cells infected with an empty LPCX retrovirus were used as controls [10]. Cells were lysed in Laemmli buffer without bromophenol blue dye.

### Real-Time Quantitative Polymerase Chain Reaction

RNA was extracted from the PC-3 and DU-145 cells treated with 0, 10, and 20 µM triol for 48 hrs using the RNeasy Minikit (cat. No. 74104, Qiagen, Venlo, The Netherlands) following the manufacturer’s instructions. The genomic DNA was removed by DNase-on-column treatment supplied with the mini kit. RNA concentration was determined spectrophotometrically at 260 nm. Equal amounts of RNA were used in the cDNA synthesis reactions using the Reverse Transciption SystemRevertAid™ H Minus First Strand cDNA Synthesis Kit (Fermentas/Thermo Scientific, Waltham, MA, USA). Real-time quantitative PCR was performed as previously described [7], [8], [21]–[23], [28] using SYBR Green system/’reagents in an optical 96-well plate and cycling conditions consisting of 2 min at 50°C, 10 min st 95°C, 40 cycles of 15 sec at 95°C, and 60 sec at 60°C on an ABI Prism system (Applied Biosystems, Foster City, CA, USA). The sequences of primers for ATP-binding cassette transporter A1 (ABCA1) were TGTCCAGTCCAGTAATGGTTC (forward) and AAGCGAGATATGGTCCGGATT (reverse). The transcript level of ABCA1 was determined in PC-3 and DU-145 cells following treatment with 0, 10, and 20 µM triol for 48 hrs and was normalized to GAPDH levels in each sample.

### Transwell Migration Assay

Migration assays with PC-3 cells were performed with a transwell kit from BD Bioscience (catalog number 353097). PC-3, DU-145, and CDXR-3 cells were treated with 0, 10, and 20 µM triol for 48 hrs. Cells were then removed from tissue culture plates with trypsin, and washed twice with PBS. Triol-treated cells (1×10^4^) in 250 ul of DMEM without serum were placed in the upper invasion chamber and the lower compartment was loaded with DMEM containing 10% FBS. The cell migration chamber was inserted into the lower compartment and incubated for either 6 (PC-3, DU-145) or 24 (CDXR-3) hrs at 37°C. Cells on the topside of the filter were removed with a cotton swab. Cells attached to the filter were then fixed with methanol for 10 min. Cells attached to the filter were then stained with Giemsa stain (5%) for 1 hour. Filters were de-stained by washing with water and the number of cells attached to the filter was then quantified by enumerating cells in photographs of the stained filters.

### Transwell Invasion Assay

An invasion assay with PC-3 cells was performed with Growth Factor Reduced BD BioCoat Matrigel invasion chambers (BD Biosciences) according to the manufacturer’s instructions. PC-3 cells were treated with different concentrations of triol for 48 hrs. Cells were then trypsinized and washed twice with PBS. Cells from each condition were seeded at 4×10^4^ cells per well in serum-free DMEM in the upper compartment, and DMEM medium with 10% FBS was placed in the lower compartment of the chamber as a chemo-attractant. After 24 hrs of incubation, the non-invading cells on the upper side of the chamber were removed, and the membranes were fixed with methanol, washed, and stained with Giemsa’s solution. Invasiveness was evaluated by counting the invading cells under a light microscope. All experiments were conducted in triplicate.

### Wound Healing Assay

CDXR-3 cells were pre-treated with 0, 10, and 20 µM triol for 24 hrs. Cells were then removed from tissue culture plates with trypsin, and washed twice with PBS. Cells were seeded at a concentration of 3.5×10^4^ cells/well in 12-well plates. After 24 hrs, a wound healing assay was performed by scraping the cells with a 200-µl pipette tip. Cells were observed at 0, 8, and 24 hrs after scraping and photographed with a live imaging microscope (Leica AF 6000 LX, Leica, Wetzlar, Germany). The migration distance was automatically measured by the program inside live imaging microscope.

### Confocal Microscopy

CDXR-3 and DU-145 cells were treated with 0 or 10 µM triol for 24 hrs. Cells were then washed 3 times with PBS buffer for 5 min per wash at room temperature (RT), fixed with 4% paraformaldehyde for 15 min at RT, and rinsed with PBS 3 times for 5 min per wash and permeabilized for 10 minutes at RT with 0.1% Triton X-100 in PBS. Cells were blocked for non-specific protein binding with 2% bovine serum albumin in PBS for 30 min, rinsed with PBS three times for 5 min each. Cells were incubated with β-actin or α-tubulin antibody for one hour. After washing, cells were incubated with secondary FITC-conjugated antibody for one hour. After washing, cells were mounted under glass slides and sealed with Permount. Images of cells were observed with an Olympus confocal microscope at 400× (ocular lens 10×; objective lens 40×) (FV300, Olympus, Tokyo, Japan).

### Data Analysis

Data are presented as the mean +/− SD of at least three experiments or are representative of experiments repeated at least three times. Student’s t test (two-tailed, unpaired) was used to evaluate the statistical significance of results from the proliferation assay experiments. A Microsoft Excel add-in program ED50V10 was used for calculating half maximal effective concentration (EC_50_).

## Results

### Triol Suppressed the Proliferation of Human Prostate Cancer Cells

**Figure 1:**
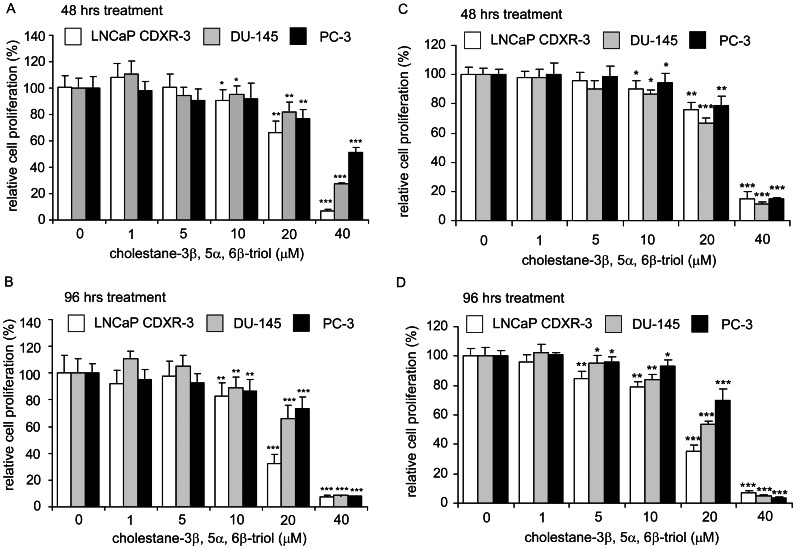
Effect of triol on viability and proliferation of human prostate cancer cell lines. LNCaP CDXR-3, DU-145, and PC-3 prostate cancer cells were treated with increasing concentrations of triol for 48 hrs (A)(C) or 96 hrs (B)(D). Relative viability and relative cell number of prostate cancer cells was determined by MTT (3,4,5-dimethylthiazol-2-yl)-2-5-diphenyltetrazolium bromide) 96-well assay (A)(B) and Hoechst dye 33258-based 96-well proliferation assay (C)(D), respectively, as described in Materials and Methods. Cell numbers were normalized to control (dimethylsulfoxide) of each cell line. Asterisk (*, **, ***) represents statistically significant difference (*p*<0.05, *p*<0.01, *p*<0.001) between the treated group and the control group.

We first sought to determine the effect of triol treatment on the viability and proliferation of three commonly used human prostate cancer cell lines (Fig. 1). LNCaP CDXR-3 cells are androgen receptor (AR)-positive, relapsed, androgen-independent cells derived from parental AR-positive androgen-dependent LNCaP 104-S cells [27]. DU-145 and PC-3 both are AR-negative androgen-insensitive cells established from brain- [30] and bone- [31] derived metastasis, respectively. An MTT assay and a Hoechst dye-based proliferation assays indicated that triol suppressed both cell viability and cellular proliferation (Fig. 1). The inhibitory effect on proliferation was dose-dependent and increased over time (Fig. 1, Table S1). The EC_50_ for triol in the MTT assay was similar to the EC_50_ measured by Hoechst dye-based proliferation assay (Table S1), suggesting that inhibition of cell proliferation was responsible for the reduction of viable cells caused by triol treatment in all three human advanced prostate cancer cell lines.

### Triol Treatment Inhibited Colony Formation of PC-3 and CDXR-3 Cells in Soft Agar

**Figure 2:**
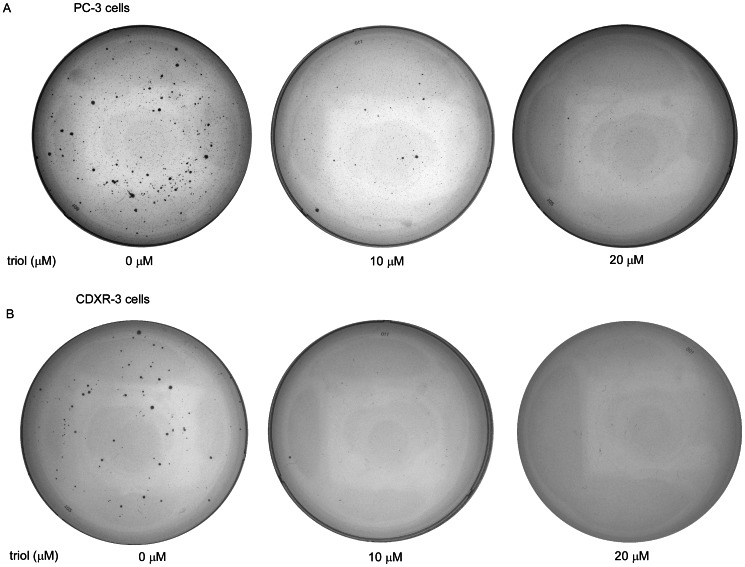
Effect of triol on colony formation in soft agar. PC-3 (A) and LNCaP CDXR-3 (B) cells treated with 0, 10, or 20 µM triol for 14 and 17 days, respectively. Image is a representative result of three biological replicates.

Treatment of PC-3 and LNCaP CDXR-3 cells with 25 µM and 50 µM triol markedly inhibited the formation of PC-3 and LNCaP CDXR-3 colonies in soft agar, confirming the anti-cancer activity of triol. DU-145 cells grew too slowly in soft agar to detect sufficient colonies for further analysis (Fig. 2).

### Triol Treatment Retarded Growth of Androgen-insensitive Prostate Cancer Xenografts in Nude Mice

**Figure 3:**
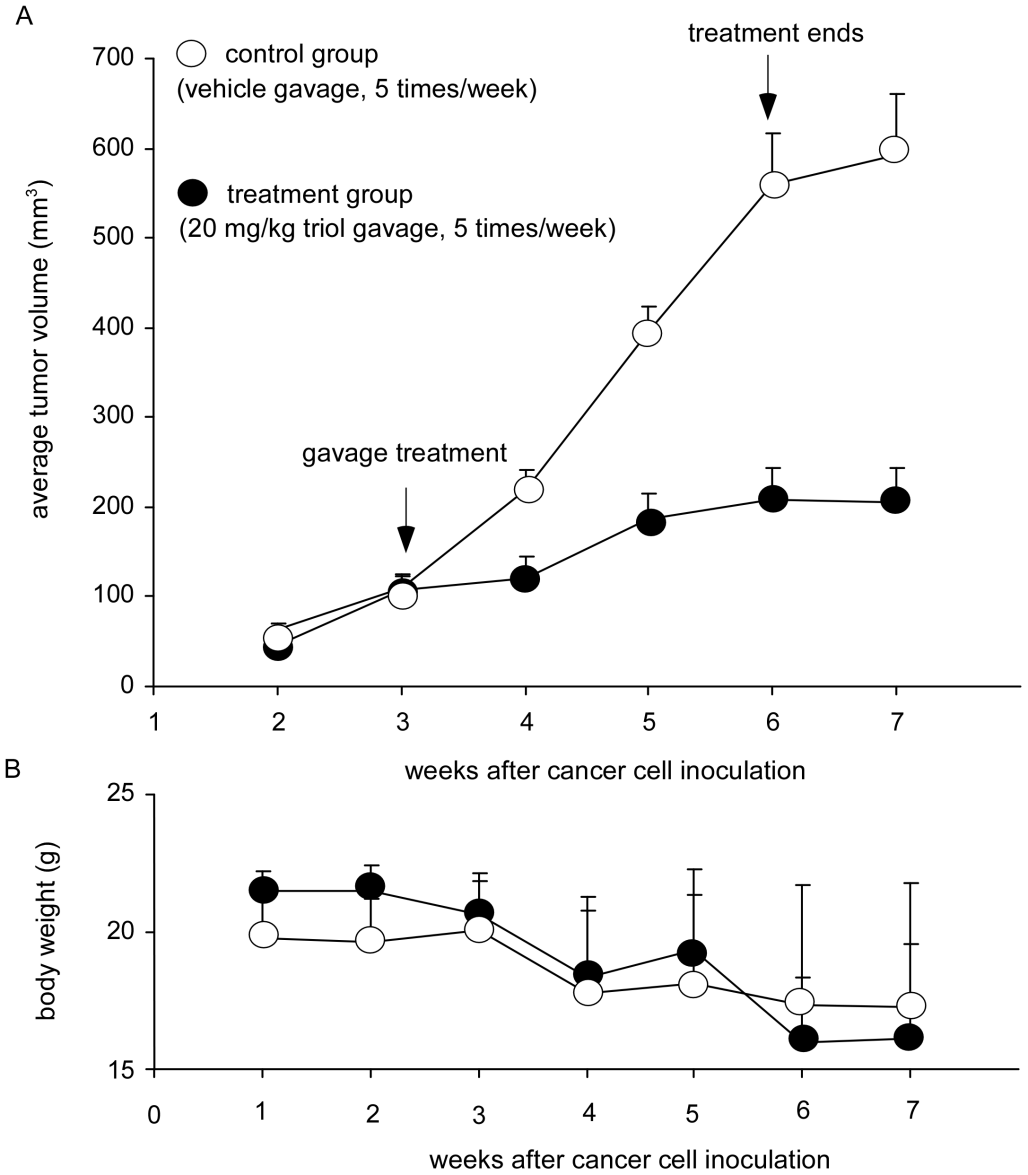
Effect of triol on the growth of PC-3 xenografts in nude mice. Male Balb/c nu/nu mice, 6–8 weeks of age, were injected subcutaneously in both flanks with 5×10^5^ PC-3 cells suspended in 0.5 ml of Matrigel. Tumors were measured daily using calipers and volume was calculated using the formula volume = length×width×height×0.52. Monitoring of tumor growth was started one week after tumor inoculation. Mice were separated into control and treatment groups. The control group had 5 mice carrying 10 tumors and the treatment group had 5 mice carrying 8 tumors. Three weeks after the initial inoculation, the treatment group was orally administrated triol daily at a dose of 20 mg/kg, 5 days/week. Mice in the control group were gavaged with vehicle only. Treatment started on day 22 and ended on day 42 after tumor inoculation. Tumor volumes were shown as mean ± standard error (A) while body weight of mice was shown as mean ± Standard Deviation (B).

To determine if triol could suppress tumor growth *in vivo*, we performed a pilot study using DU-145 xenografts in nude mice. Intraperitoneal (i.p.) injection of 1 mg (50 mg/kg) of triol daily for 14 days caused a 36% reduction in the average volume of DU-145 xenografts growing in nude mice (Fig. S1). Triol treatment for 14 days did not affect the body weight of the mice (data not shown). To determine if lower oral doses of triol could inhibit the growth of PC-3 prostate xenografts. we orally administered triol at 20 mg/kg five times a week. Oral administration of 20 mg/kg triol (5 times per week) from the 3^rd^ to 6^th^ week caused a 65% reduction in average tumor volume (p = 0.0002) in the treatment group compared to tumor volume in the vehicle control group (Fig. 3A). Body weight of both vehicle and triol-treated groups gradually decreased (Fig. 3B). This may be due to cachetic effects of PC-3 xenografts. These observations confirmed that oral administration of triol could retard the growth of prostate tumors *in vivo*.

### Triol Treatment Caused G1 Cell Cycle Arrest in Prostate Cancer Cells

**Figure 4:**
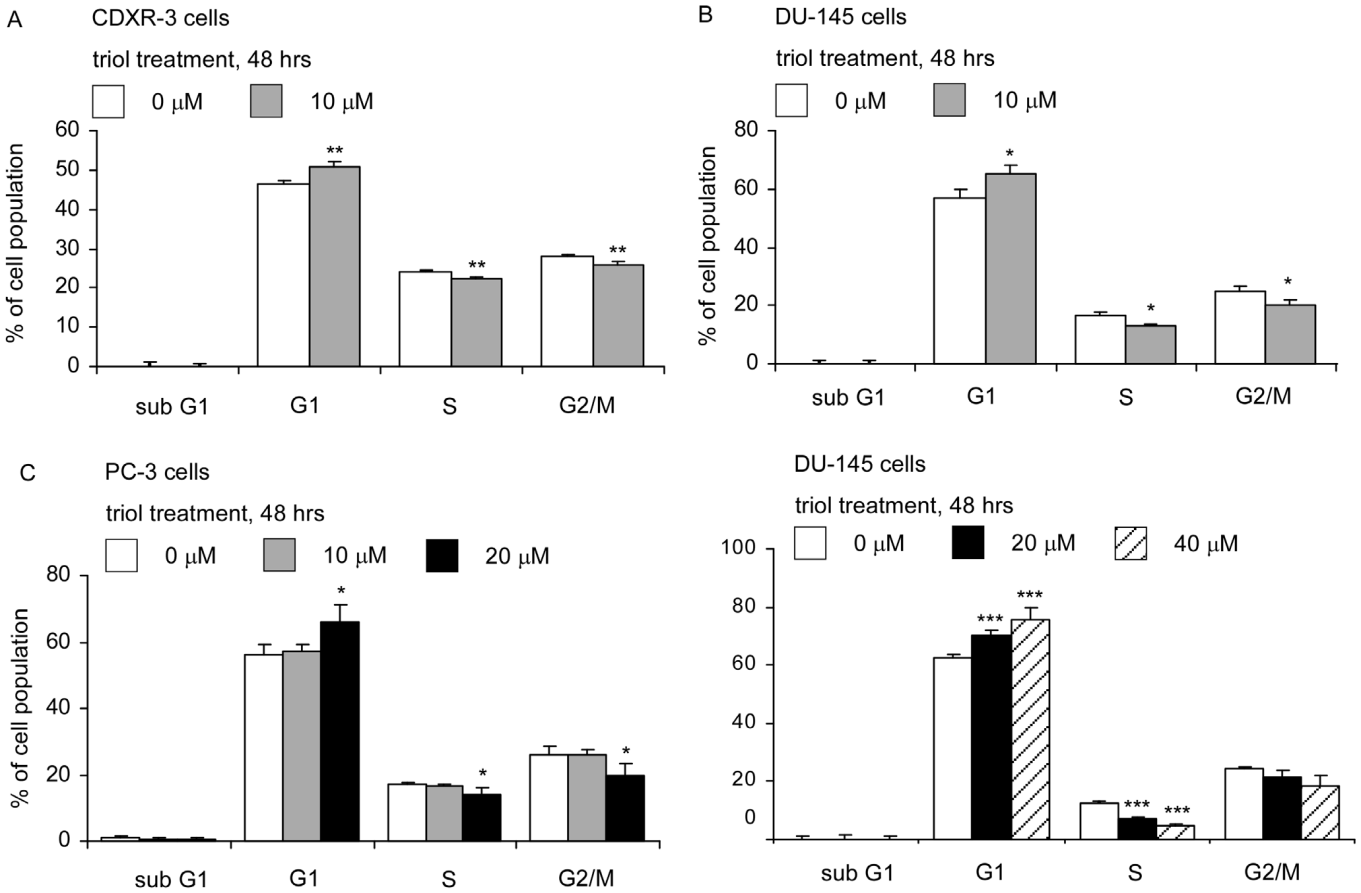
Effect of triol on cell cycle distribution of prostate cancer cells. LNCaP CDXR-3 (A) and DU-145 cells (B) were treated with 0 or 10 µM triol for 48 hrs. PC-3 cells (C) were treated with 0, 10, or 20 µM triol for 48 hrs. (D) DU-145 cells were treated with 0, 20, and 40 µM triol for 48 hrs. Cell cycle distribution was determined by flow cytometry. Asterisk (*, **, ***) represents statistically significant difference (*p*<0.05, *p*<0.01, and *p*<0.001, respectively) between the treated group and the control group.

We next performed flow cytometric analysis to determine if cell cycle progression of prostate cancer cells was affected by triol. Treatment with 10 µM triol for 48 hrs caused an increase in the G1 phase cell population and a decrease in S and G2/M phase populations of LNCaP CDXR-3 and DU-145 prostate cancer cells (Fig. 4A, 4B). Triol at 10 µM did not affect cell cycle progression of PC-3 cells. However, 20 µM triol caused a significant increase in G1 and decrease in the S and G2/M populations of PC-3 cells (Fig. 4C). Therefore, treatment with 10–20 µM triol induced G1 cell cycle arrest in LNCaP, DU-145, and PC-3 cells. We did not observe any increase in sub-G1 population in these three prostate cancer cell lines. To determine if higher concentrations of triol will augment the population of cells in sub-G1, we treated DU-145 cells with 0, 20, and 40 µM triol (Fig. 4D). Reduction of S phase population and induction of G1 phase population of DU-145 cells was even more significant after treatment with 40 µM triol. However, there was no significant increase of cells in the sub-G1 population.

### Treatment with High Concentration of Triol Induced Apoptosis in Prostate Cancer Cells

**Figure 5:**
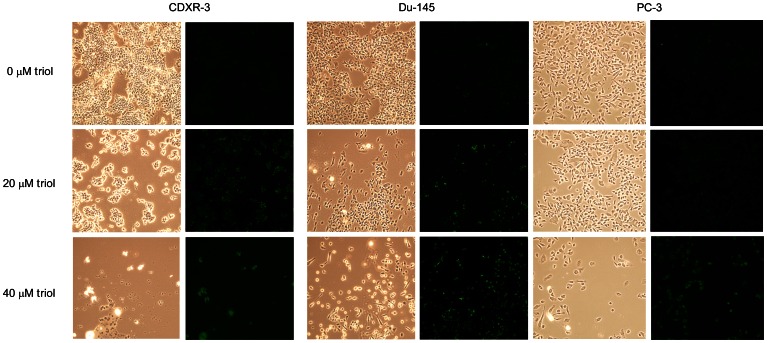
Triol induces apoptosis in prostate cancer cells. **LNCaP CDXR-3, DU-145, and PC-3 cells were treated with 0, 20, and 40 µM triol for 48 hrs.** Cell morphology was determined by light microscopy. TUNEL assay was performed as described in Materials and Methods to determine the apoptotic cell population. Green fluorescent light indicated apoptotic cells stained with TUNEL assay. Images were viewed at 200× with Olympus confocal microscope.

As propidium iodide staining flow cytometric analysis is not a reliable method to detect apoptosis, we used the TUNEL assay to determine if triol treatment at higher concentrations induced apoptosis in prostate cancer cells. We treated CDXR-3, DU-145, and PC-3 cells with 0, 20, and 40 µM triol for 48 or 96 hrs. Consistent with the flow cytometry analysis data, treatment with triol for 48 hrs only produced a small population of apoptotic cells in all these cell lines (data not shown). Treatment with triol for 96 hrs dose-dependently resulted in an increased the population of apoptotic cells in all three prostate cancer cell lines (Fig. 5). Although treatment with 20 µM triol for 96 hrs only slightly increased the apoptotic population, treatment with 40 µM triol resulted in a significant increase in apoptosis in all three prostate cancer cell lines.

### Micro-Western Array Revealed Signaling Proteins being Affected by Triol Treatment

**Figure 6:**
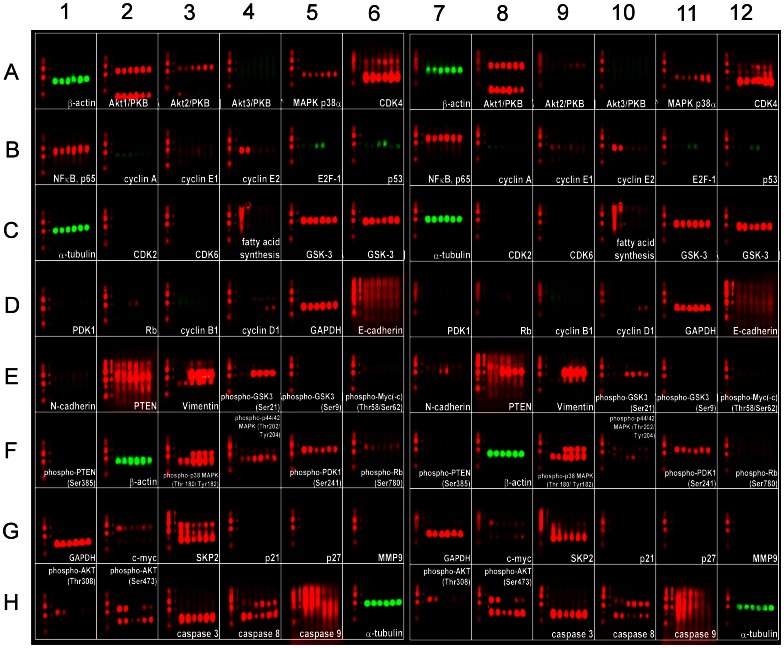
Micro-Western Array analysis of protein expression in CDXR-3, DU-145, and PC-3 cells treated with 0 or 20 µM triol. CDXR-3, DU-145, and PC-3 cells were treated with 0 or 20 µM of triol for 48 hrs. Cell lysates were collected according to Micro-Western Array (MWA) protocol [18] and MWAs were performed to measure the changes in abundance and modification of indicated proteins. The six samples printed in each well from left to right were CDXR-3 (control), CDXR-3 (20 µM triol), DU-145 (control), DU-145 (20 µM triol), PC-3 (control), PC-3 (20 µM triol) (Figure S2). The right half blot (well A7-H12) was the technical duplicate of the left half blot (well A1-H6). Antibody list is shown in Table S2. Red and green were artificial color for anti-rabbit and anti-mouse 2^nd^ antibody, respectively. The blot is the same size as a standard 96-well microtiter plate. Relative protein abundance of each signaling protein in each cell line is listed in Table S3.

**Figure 7:**
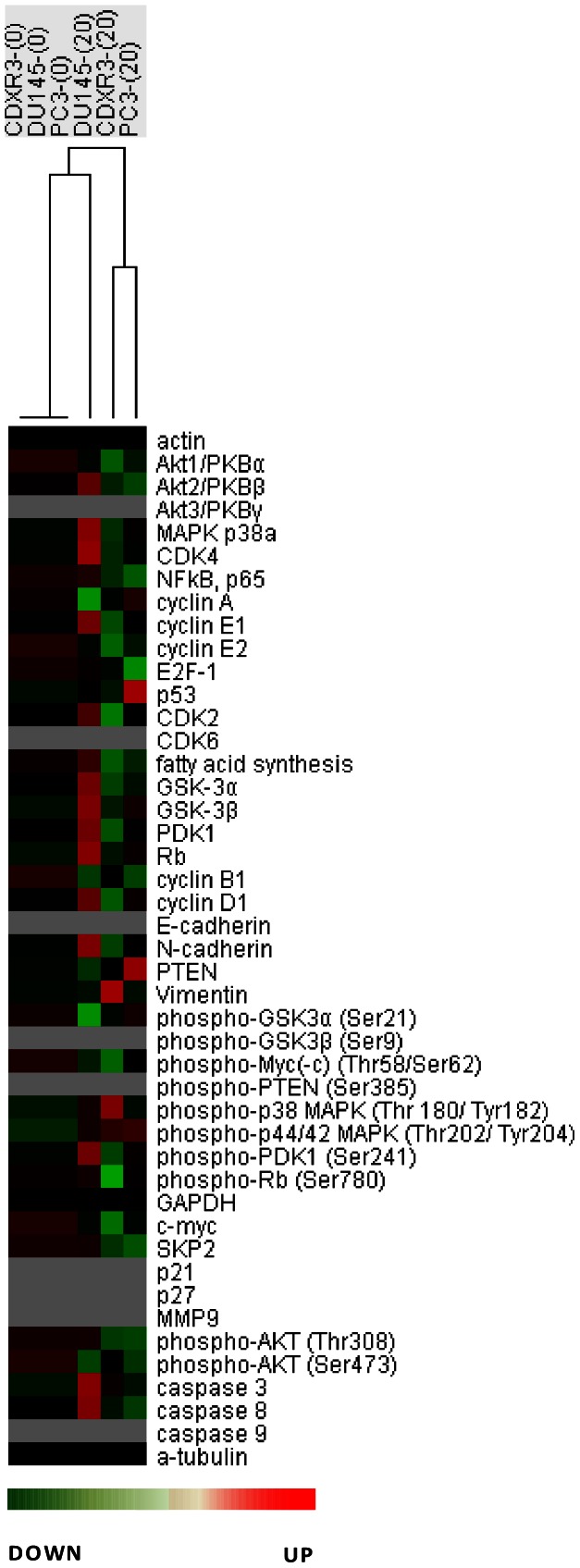
Protein expression profile of Micro-Western Array results. Protein expression profiles of CDXR-3, DU-145, and PC-3 cells treated with triol are displayed in a heat map format. Black color indicated no change, while green and red colors indicated decrease and increase of protein expression level. Grey color indicated signal below detection threshold. Protein abundance was normalized to the average of α-tubulin, GAPDH, and β-actin. Proteins in different cell lines were normalized to the control condition (no triol treatment) of that cell line.

We hypothesized that triol may reduce cell viability through its effect on protein signaling networks. In order to determine what signaling proteins might be affected by triol treatment, we used Micro-Western Arrays (MWAs), a high-throughput Western blotting assay [17]–[20], [24], to screen for signaling proteins affected by treatment with 10 µM triol in CDXR-3 and DU-145 cells. PTEN is frequently deleted in prostate cancer, resulting in activation of PI3K/Akt signaling [32]. PI3K/Akt signaling plays an important role in survival and progression of prostate cancer cells [32], [33]. Up-regulation of PI3K/Akt activity is associated with poor clinical outcome of prostate cancer [34]. We used 48 antibodies that are capable of detecting proteins regulating cell cycle progression, cell survival and apoptosis, Akt-related signaling pathways, and several epithelial-mesenchymal transition (EMT) markers (Figure S2, Table S2) for the screening portion of our MWA study (Fig. 6). Differences in the protein expression profile in the absence and presence of 20 µM triol is shown in Fig. 7. Each of the three cell lines had a unique protein expression response to triol treatment. The protein profiles of CDXR-3 and PC-3 cells following treatment with 20 µM triol were more similar to each other than to DU-145 cells. Triol caused reduction of Akt1, cyclin E2, cyclin B1, phospho-c-Myc Thr58/Ser62, c-Myc, and phospho-Akt Ser473 in all three prostate cancer cell lines. Triol treatment at 20 µM resulted in at least a 20% increase in the protein abundance of phospho-p38 MAPK Thr180/Tyr182 (CDXR-3), CDK4 (DU-145), cyclin E1 (DU-145), cyclin D1 (DU-145), and p53 (PC-3); but caused at least a 20% decrease in the protein abundance of cyclin D1 (CDXR-3), phospho-c-Myc Thr58/Ser62 (CDXR-3), phospho-Rb Ser780 (CDXR-3), c-Myc (CDXR-3, PC-3), phospho-Akt Thr308 (CDXR-3, PC-3), phospho-Akt Ser473 (DU-145, PC-3), cyclin A (DU-145), cyclin B1 (DU-145, PC-3), phospho-GSK3α Ser21 (DU-145), NF-κB p65 (PC-3), and fatty acid synthase (FASN) (PC-3) (Table S3).

### Triol Decreased the Level or Phosphorylation Status of Signaling Proteins Involved in Cell Cycle and Akt Signaling

**Figure 8:**
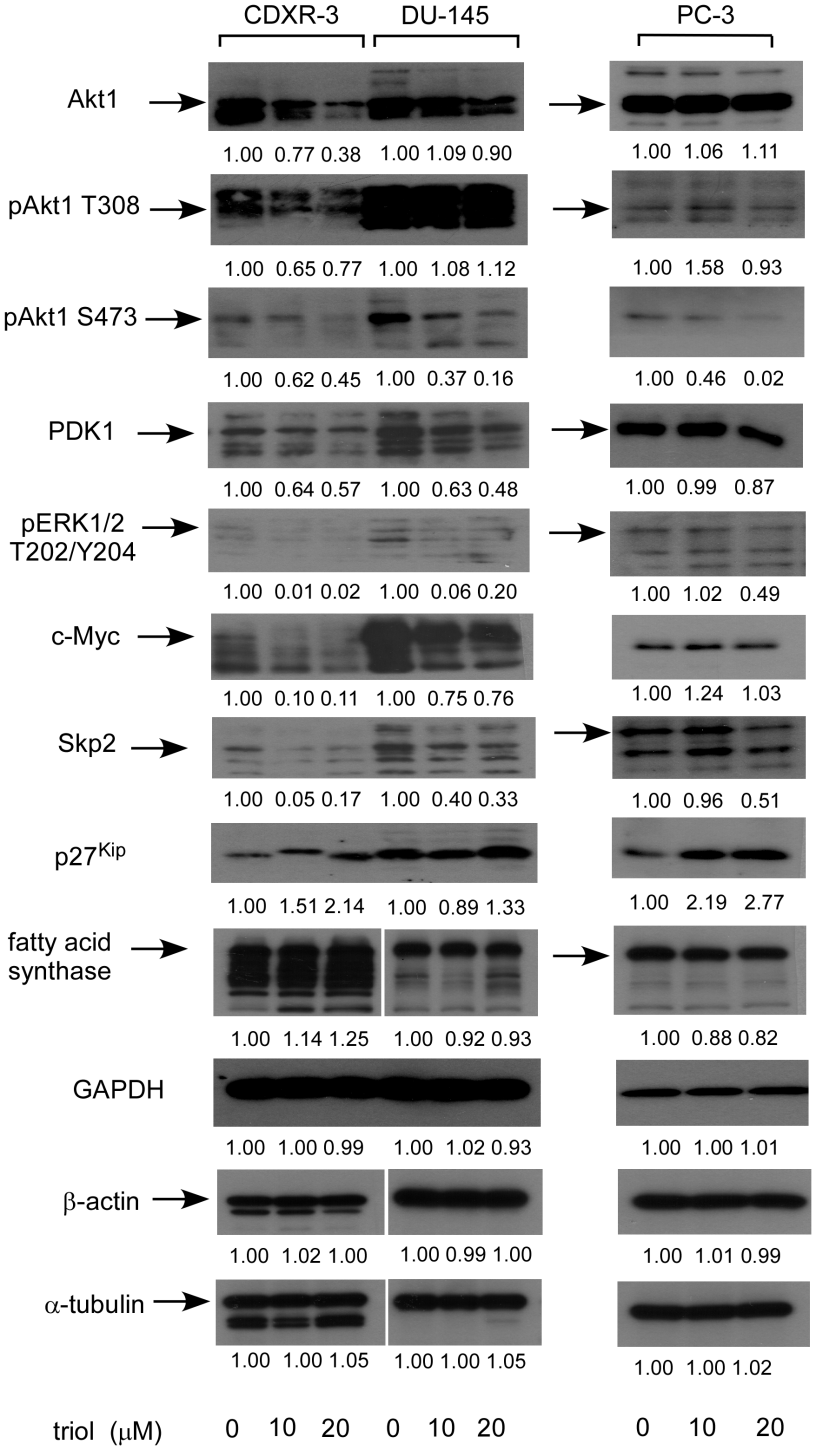
Effect of triol treatment on the abundance and phosphorylation status of signaling proteins. Protein expression of Akt1, phospho-Akt Thr308, phospho-Akt Ser473, PDK1, phospho-p44/42 MAPK Thr202/Tyr204, c-Myc, Skp2, p27^Kip1^, FASN, GAPDH, β-actin, and α-tubulin in CDXR-3, DU-145, and PC-3 cells treated with 0, 10 or 20 µM triol for 48 hrs were assayed by Western blotting. Values represent relative protein abundance.

The MWA result suggested that triol treatment altered the abundance and phosphorylation status of signaling proteins involved in cell cycle and Akt signaling in prostate cancer cells. We performed conventional Western blotting analysis to confirm these results. Triol treatment caused a decrease of Akt1 (DU-145, CDXR-3), phospho-Akt Thr308 (DU-145), phospho-Akt Ser473 (DU-145, CDXR-3, PC-3), PDK1 (DU-145, CDXR-3, PC-3), phospho-p44/42 MAPK Thr202/Tyr204 (DU-145, CDXR-3, PC-3), c-Myc (DU-145, CDXR-3), and S-phase kinase-associated protein 2 (Skp2) (DU-145, CDXR-3, PC-3). Triol treatment also resulted in elevated cell cycle inhibitor p27^Kip^ in all three cell lines. FASN expression was slightly increased in DU-145 but slightly decreased in CDXR-3 and PC-3 cells treated with triol (Fig. 8).

### Overexpression of Skp2 Partially Rescued the Inhibitory Effect of Triol Treatment in PC-3 Cells

**Figure 9:**
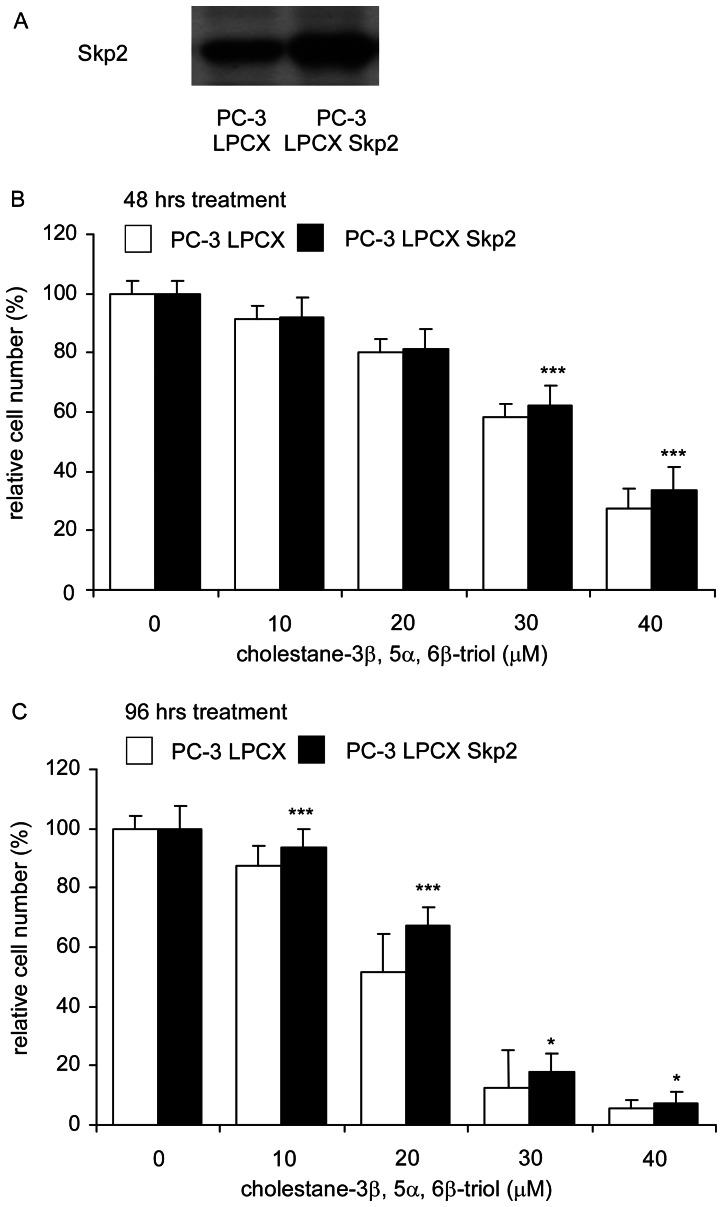
Effect of overexpression of Skp2 protein on growth inhibition by triol treatment. (A) Protein expression of Skp2 assayed by Western blotting in PC-3 LPCX control and PC-3 LPCX Skp2 cell lines. PC-3 cells overexpressing Skp2 with LPCX vector and control PC-3 cells (empty LPCX vector) were treated with increasing concentrations of triol for 48 hrs (B) or 96 hrs (C) and analyzed by the 96-well proliferation assay. Asterisk * and *** represent statistically significant difference (*p*<0.05 and *p*<0.001) between the PC-3 control cells and PC-3 overexpressing Skp2- cells.

Since triol treatment caused a significant decrease in Skp2 expression and an increase in p27^Kip^ expression in all three prostate cancer cell lines (Fig. 8), we next wished to determine if overexpression of Skp2 would block the growth inhibition caused by triol in PC-3 cells. Indeed, overexpression of Skp2 partially rescued the cell proliferation rate of PC-3 cells under triol treatment (Fig. 9). Because overexpression of Skp2 did not completely reverse the growth inhibitory effects, we hypothesized that other additional pathways or mechanisms may be influenced by triol to cause cell growth inhibition of prostate cancer cells.

### Triol Treatment Suppressed Akt1 in PC-3 Xenograft

**Figure 10:**
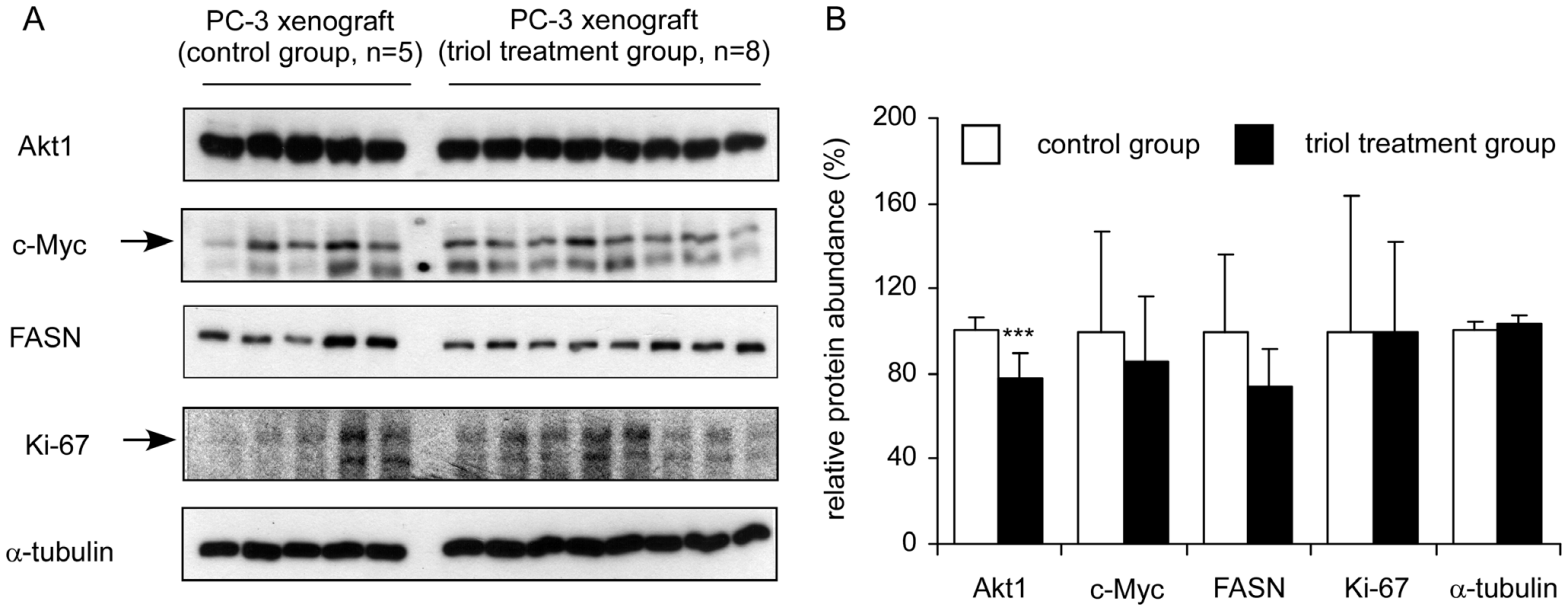
Triol treatment affected expression of Akt1 protein in PC-3 xenografts. (A) Protein expression of Akt1, c-Myc, FASN, Ki-67, and α-tubulin was examined in 5 PC-3 tumors from control group and 8 PC-3 tumors from treatment group (Fig. 3). (B) Relative protein abundance of each signaling protein was quantified and calculated for average and standard deviation. Asterisk *** represents statistically significant difference (*p*<0.001) between the control and triol treatment group.

We examined the protein expression of Akt1, c-Myc, FASN, Ki-67, and α-tubulin in 5 tumors xenografts from the vehicle-treated group and 8 tumor xenografts from the triol-treated group from Fig. 3. Triol treatment resulted in significantly reduced Akt1 expression in PC-3 xenografts (*p*<0.001, Fig. 10). Tumors in the treatment group tended to express lower c-Myc and FASN levels, although the p value was larger than 0.05. We also determined the protein levels of E-cadherin, N-cadherin, MMP-9, vimentin, slug, snail, p27^Kip^, and phospho-Akt Ser473 in these xenografts. The variation between individual tumors within both control and treatment groups of these proteins was large and no significant difference in specific protein abundances between control and treatment groups (data not shown).

### Triol is as a Weak Liver X Receptor (LXR) Agonist

**Figure 11:**
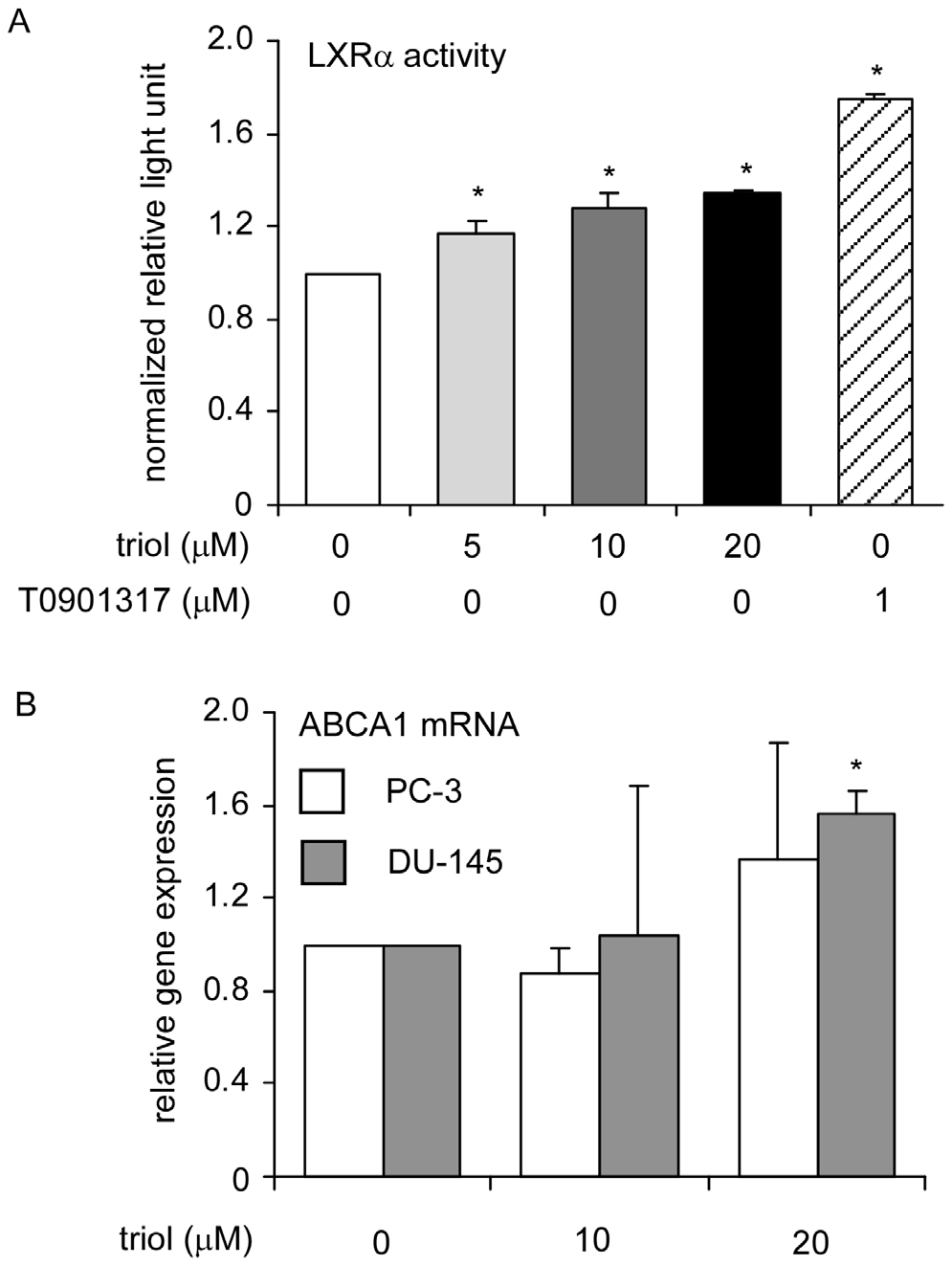
Effect of triol on LXR reporter and ABCA1 gene expression. (A) PC-3 cells were transfected with pRL-TK-Renilla luciferase plasmid, pSG5RXRα, pSG5LXRα, and 4xDR4Δ56cfos pGL3 reporter gene plasmid. PC-3 cells were treated with 0, 5, 10, 20 µM triol or 1 µM T0901317. Luciferase reporter gene assay was performed to determine the activity of LXRα receptor. (B) PC-3 and DU-145 cells were treated with 0, 10, 20 µM triol for 48 hrs. ABCA1 gene, a target gene of LXRα, was determined by qRT-PCR. Asterisk * represents statistically significant difference (*p*<0.05) between the control and triol treatment group.

LXRs are ligand-activated transcriptional factors that belong to the nuclear receptor super family. LXRs are important regulators of cholesterol, fatty acid, and glucose homeostasis [9]. Two LXR isoforms exist: LXRα expression is most abundant in liver, kidney, intestine, fat tissue, macrophages, lung, and spleen; LXRβ is expressed ubiquitously [9]. A specific group of oxysterols, including 22(R)-hydroxycholesterol, 20(S)-hydroxycholesterol, 24(S)-hydroxycholesterol, and cholestenoic acid are natural ligands for LXRs [9], [10]. We previously reported that LXR agonists, such as T0901317, 22(R)-hydroxycholesterol, and 24(S)-hydroxycholesterol suppress the proliferation of prostate and breast cancer cells via induction of G1 cell cycle arrest [6]–[10]. Since triol is an oxysterol, it might produce some effects as an agonist for LXR. To determine if triol is an LXR agonist, we determined if triol could activate LXR and induce expression of a luciferase reporter gene containing a LXR response element. Triol only slightly increased the transcriptional activity of LXRα (Fig. 11A). However, compared to the well known LXR agonist T0901317, triol was a very weak LXRα agonist (Fig. 11A). Treatment of DU-145 cells with 20 µM triol resulted in a slight increase in expression of ABCA1 mRNA expression, a target gene of LXRα activity (Fig. 11B), confirming the agonistic activity of triol for LXRα. The agonistic activity of triol for LXRα may partially contribute to the growth inhibition and G1 cell cycle arrest of prostate cancer cells.

### Triol Treatment Altered Actin and Tubulin Distribution in Prostate Cancer Cells

**Figure 12:**
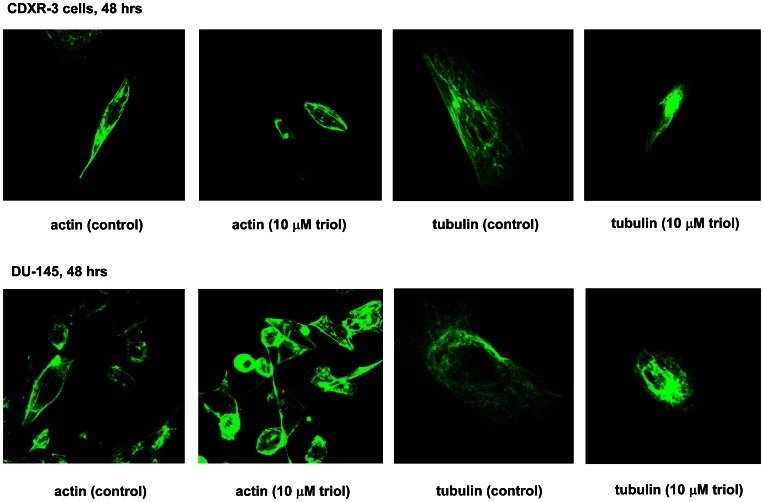
Effect of triol treatment on cytoskeleton morphology in CDXR-3 and DU-145 cells. Imaging of α-tubulin and β-actin in CDXR-3 and DU-145 cells treated with vehicle or 10 µM triol for 48 hrs was determined by confocal microscopy.

Triol was previously reported to cause disruption of actin microfilaments [35], redistribution of vimentin [36], and loss of vinculin [35] in 73/73 endothelial cells. We next sought to determine if the cytoskeleton was affected by triol treatment of prostate cancer cells. Confocal laser microscopy revealed a modification of the β-actin and α-tubulin microfilaments, especially redistribution at the periphery of the cells following 48 hrs of treatment with 10 µM triol in CDXR-3 and DU-145 cells (Fig. 12). We did not observe significant changes in β-actin and α-tubulin microfilaments in PC-3 cells treated with 10 µM triol (data not shown).

### Triol Treatment Suppressed Migration and Invasion of Prostate Cancer Cells

**Figure 13:**
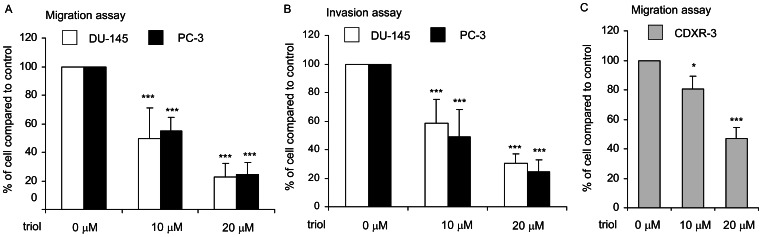
Effect of triol treatment on migration and invasion of PC-3, DU-145, and CDXR-3 cells. PC-3, DU-145, and CDXR-3 cells were pre-treated with 0, 10, or 20 µM triol for 48 hrs. Cells were then counted and seeded at identical number. Cancer cell migration ability (A) and invasion ability (B) was determined by transwell migration assay (A) and transwell invasion assay 6 hrs (A) and 24 hrs (B) after cell seeding, respectively. (C) Migration of CDXR-3 cells was determined by transwell migration assay 24 hrs after cell seeding. There was no triol in medium during the migration or invasion assay. Asterisk *** represents statistically significant difference (*p*<0.001) between the control group and triol treatment group.

**Figure 14:**
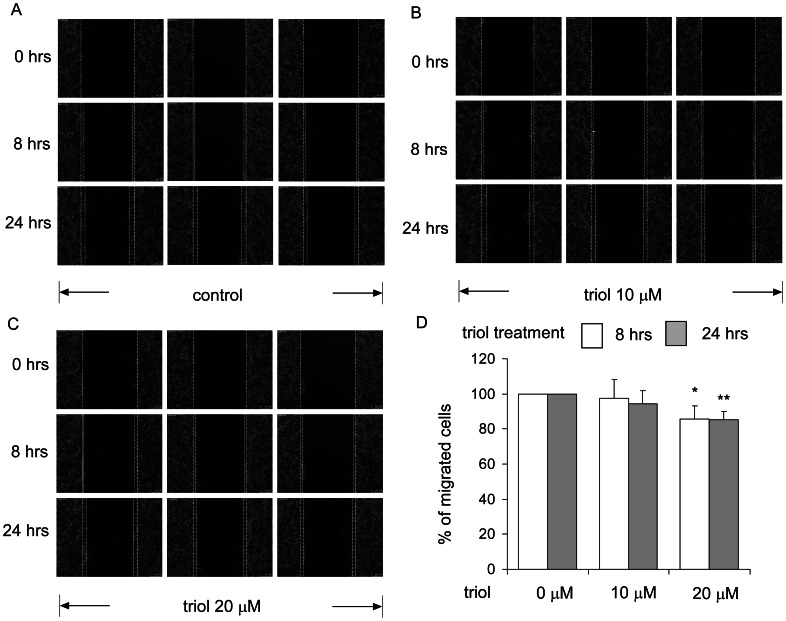
Triol treatment inhibited migration of CDXR-3 cells determined by wound healing assay. Wound healing assay was performed to determine the effect of (A) 0 µM (B) 10 µM or (C) 20 µM triol on migration ability of CDXR-3 cells 8 or 24 hrs after cell seeding. (D) Results of (A)(B)(C) were quantified. Asterisk * and ** represents statistically significant difference (*p*<0.05 and *p*<0.01) between the control group and the triol treatment group.

Since triol resulted in changes in the structure and morphology of the cytoskeleton, we suspected that triol treatment might affect the migration and invasion of prostate cancer cells. Pre-treatment of prostate cancer cells with 10 µM or 20 µM triol for 48 hrs significantly inhibited the migration and invasiveness of DU-145 and PC-3 cells (Fig. 13A, 13B). LNCaP cells have poor migration and invasion ability. Extending the time for transwell migration assay revealed that migration of LNCaP CDXR-3 was suppressed dose-dependently by triol treatment (Fig. 13C). A wound healing assay confirmed that treatment with 20 µM triol significantly reduced the migration ability of LNCaP CDXR-3 cells (Fig. 14).

### Triol Treatment Affected Epithelial-mesenchymal Transition Proteins in Prostate Cancer Cells

**Figure 15:**
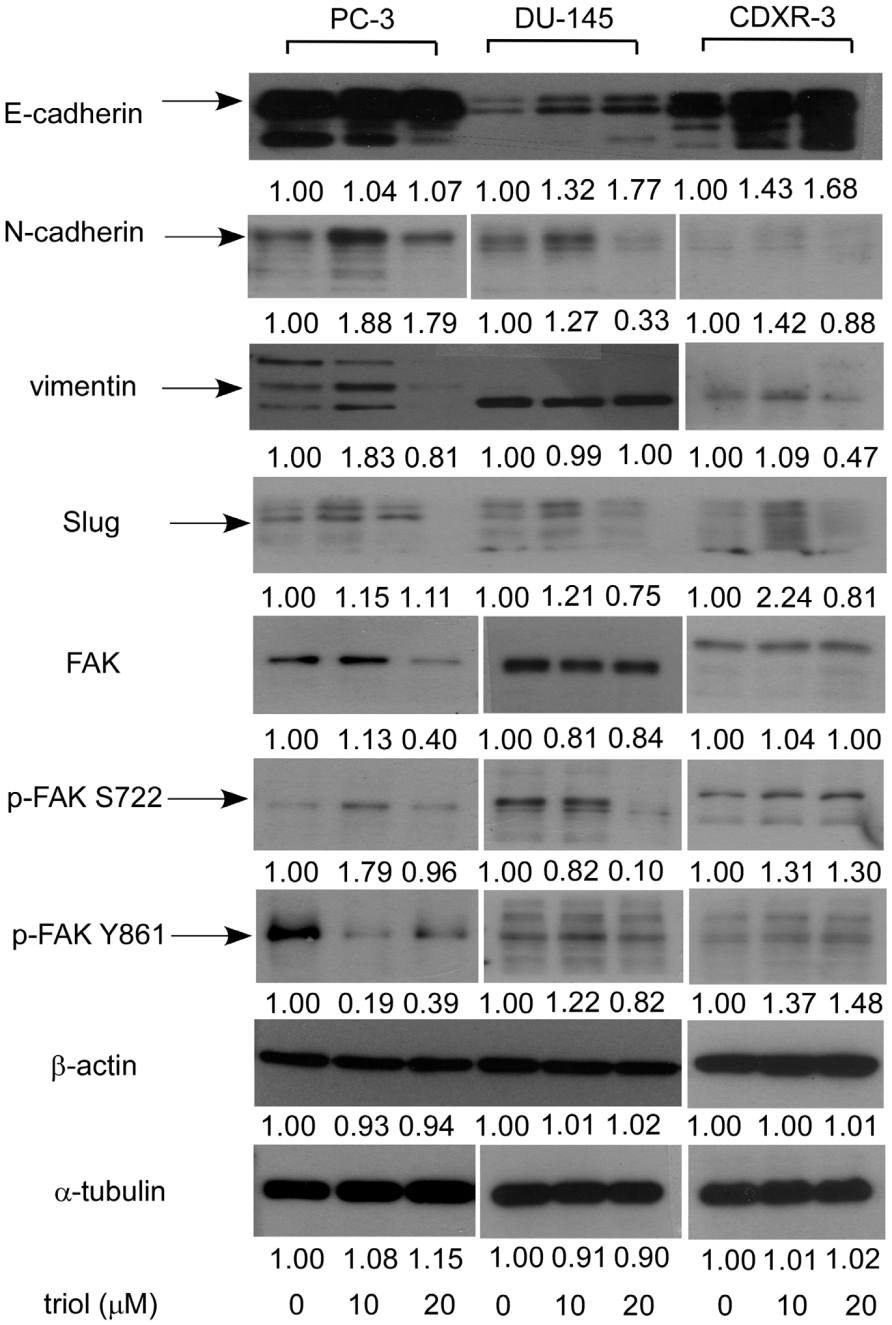
Effect of triol on expression levels of EMT marker and FAK proteins in PC-3, DU-145, and CDXR-3 cells. Protein expression of E-cadherin, N-cadherin, vimentin, Slug, FAK, phospho-FAK Ser722, phospho-FAK Tyr861, β-actin, and α-tubulin in PC-3, DU-145, and CDXR-3 cells treated with 0, 10, or 20 µM triol for 48 hrs were assayed by Western blotting. Values represent relative protein abundance.

Epithelial-mesenchymal transition (EMT) marker proteins regulate the ability of cancer cells to migrate and invade. We determined if triol treatment altered the expression level of these EMT marker proteins. Triol treatment (20 µM) resulted in increased E-cadherin protein abundance (DU-145, CDXR-3), N-cadherin (PC-3 cells), and phospho-focal adhesion kinase (FAK) Ser722/Tyr861 (CDXR-3) cells. Triol treatment (20 µM) resulted in decreased protein levels of N-cadherin (DU-145, CDXR-3), vimentin (PC-3, CDXR-3), Slug (DU-145, CDXR-3), FAK (PC-3, DU-145), phospho-FAK Ser722 (DU-145), and phospho-FAK Tyr861 (PC-3, DU-145). (Fig. 15).

### Non-cancerous Human Prostate Epithelial Cells were More Resistant to Triol Treatment

**Figure 16:**
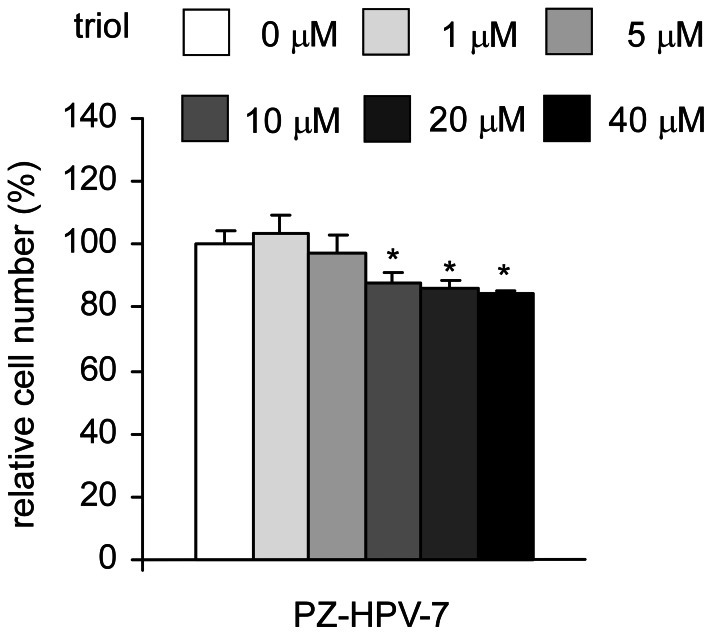
Effect of triol on viability of non-cancer human cell lines. Human prostate epithelial PZ-HPV-7 cells were treated with increasing concentrations of triol for 48 hrs. Relative cell viability was determined by MTT assay as described in Material and Methods. Cell numbers were normalized to control (dimethylsulfoxide treatment) for each cell line. Asterisk * represents statistically significant difference (*p*<0.05) between the treated and the control group.

We sought to determine the effect of triol on the proliferation of non-cancerous human cells. In place of normal prostate epithelial cells, we used PZ-HPV-7 cells. PZ-HPV-7 cells were derived from normal human prostate epithelial cells transformed by transfection with HPV18 DNA. Compared to the three prostate cancer cell lines in this study, PZ-HPV-7 cells were more resistant to triol treatment. After 48 hrs treatment with triol, the EC_5_0 for triol suppression of proliferation of PZ-HPV-7 cells was 110.5 µM (Fig. 16). This suggested the possibility that non-cancerous human prostate cells may be more resistant to triol treatment compared to prostate cancer cells. However, the higher resistance of PZ-HPV-7 cells to triol treatment might be due to the lower proliferation rate of these non-cancerous cells compared to that of cancer cells. Further studies are needed to determine if triol will adversely affect normal prostate cells.

## Discussion

Oxysterols are more polar and more readily diffusible through cell membranes than cholesterol, and possesses higher pro-inflammatory and cytotoxic effects than cholesterol in certain cells and tissues [13], [37]. Oxysterols have been reported to be potentially involved in the initiation and progression of atherosclerosis, neurodegeneration, diabetes, kidney failure, and ethanol intoxication [13]. In cholesterol-rich foods, the most commonly found oxysterols include 7α-hydroxycholesterol, 7β-hydroxycholesterol, 7-ketocholesterol, 5α,6α-epoxycholesterol, and 5β,6β-epoxycholesterol [13]. Both 5α, 6α-epoxycholesterol and 5β, 6β-epoxycholesterol are unstable and are converted to cholestane-3β, 5α, 6β-triol [15], [16]. Oxysterols have been shown to suppress the proliferation and survival of human cancer cells *in vitro* [7]–[14]. The cytotoxicity of oxysterols may cause growth inhibition and cell death in cancer cells if applied locally to tumors.

We observed that triol treatment inhibited tumor growth, reduced cell proliferation, induced G1 cell cycle arrest, and altered cytoskeletal morphology of human prostate cancer cells. Oral administration of triol at 20 mg/kg for three weeks reduced the average tumor volume of AR-negative androgen-insensitive PC-3 prostate cancer cells by 65% (Fig. 3). PC-3, one of the most commonly used prostate cancer cell lines, was established from bone-derived metastases [31]. Prostate cancer has a predilection to metastasize to bone and more than 80% of patients who died from prostate cancer developed bone metastases [38]–[40]. Our animal study suggested that triol treatment might be a potential treatment for prostate cancer metastasis. Indeed, we demonstrated that triol suppressed migration and invasion of DU-145, PC-3, and LNCaP CDXR-3 cells. The inhibition of migration and invasion of prostate cancer cells was not due to reduction of cell proliferation because the triol was given as a pre-treatment only and the same number of prostate cancer cells was seeded in both control and pre-treatment groups for the transwell assay (Fig. 9). Cancer cells in certain circumstance recapitulate normal developmental processes to enhance cell migration and invasion, a procedure called epithelial-to-mesenchymal transition (EMT). During the EMT, polarity is lost, motility is increased, mesenchymal markers are up-regulated, and differentiation markers are down-regulated [41], [42]. This process was linked to cancer progression, in which epithelial cells acquire increased capacity for migration, stromal invasion, and metastasis [41], [42]. During EMT of prostate cancer cells, the expression of transcription factors such as Snail, Slug, Twist, E47, ZEB1, and ZEB2 is increased, which leads to increased expression of N-cadherin and vimentin and concomitantly decreased expression of E-cadherin and cytokeratins [42]–[45]. Our study suggested that triol treatment resulted in decreased abundance of the proteins Slug, vimentin, and N-cadherin proteins while resulting in increased expression of the protein E-cadherin protein (Fig. 10). Focal adhesion kinase (FAK) is a non-receptor tyrosine kinase localized in focal contacts that becomes tyrosine phosphorylated. FAK signaling pathway is involved in the regulation of prostate cancer cell migration [46]–[49]. We observed that triol treatment resulted in reduced abundance of FAK in PC-3 cells as well as phospho-FAK Ser722 in DU-145 cells and phospho-FAK Tyr861 in PC-3 cells. This new finding indicated that triol treatment targeted both EMT markers and FAK signaling proteins and can thus be a potential therapeutic agent for treatment of prostate cancer metastasis. We observed that non-cancerous human prostate epithelial PZ-HPV-7 cells were more resistant to triol treatment compared to LNCaP, DU-145, and PC-3 prostate cancer cells (Fig. 11). This result suggested that triol has selectivity against prostate cancer cells versus normal cells.

Micro-Western Arrays (MWAs) represent a new method for proteomic analyses [19]. The MWA is a modification of the antibody-based modified reverse phase protein array. A non-contact microarray (such as the GeSim Nanoplotter), a flatbed electrophoresis apparatus (such as the GE multiphor), and a near infrared scanner (such as the Licor Odyssey) comprise the MWA analysis system [18]. The MWA system allows detection of the abundance or modification status of up to 384 different proteins with as many as 15 samples simultaneously. The method has been shown to be effective in characterizing the EGFR pathway under multiple growth factor concentrations and in network modeling of complex receptor tyrosine kinase (RTK) cross-talk after stimulation [18]. The MWA has a technical advantage over other current technologies for assaying protein modifications such as Reverse Phase Lysate Arrays, quantitative Mass Spectroscopy, and Luminex through its ability to efficiently and accurately measure hundreds of chosen phosphosites simultaneously, expanding the number of pathways surveyed in a single experiment. We applied this technology to screen signaling proteins being affected by triol in prostate cancer cells. In our MWA screening using 48 antibodies against proteins involved in Akt signaling and cell cycle regulation, we observed that triol caused reduction of Akt1, Akt2, cyclin E2, cyclin B1, phospho-c-Myc Thr58/Ser62, c-Myc, and phospho-Akt Ser473 in all three prostate cancer cell lines. Treatment with 20 µM triol caused at least a 20% increase in protein abundance of the following proteins: phospho-p38 MAPK Thr180/Tyr182, CDK4, cyclin E1, cyclin D1, and p53; while triol decreased in protein expression of cyclin D1, phospho-c-Myc Thr58/Ser62, phospho-Rb Ser780, c-Myc, phospho-Akt Thr308, phospho-Akt Ser473, cyclin A, cyclin B1, phospho-GSK3α Ser21, NF-κB p65, and FASN (Table S3). These findings were confirmed by conventional Western blotting. Western blotting experiments also showed that the protein levels of Akt1, phospho-Akt Ser473, phospho-Akt Thr308, PDK1, c-Myc, Skp2 in prostate cancer cells was decreased by triol treatment (Fig. 8). PI3K/Akt signaling plays an important role in survival and progression of prostate cancer cells [32], [33], [50]. Akt is a serine/threonine protein kinase encoded in mammals by three unique genes: Akt1, Akt2, and Akt3 [51], [52]. Akt1 is involved in cellular survival pathways and inhibits apoptotic processes. Akt contains at least two important phosphorylation sites: threonine 308 and serine 473, which regulate its activity. Phosphorylation of threonine 308 on Akt is activated by PDK1 [53]. Phosphorylation of serine 473 is activated by mTOR kinase, its associated protein rictor, and SIN1/MIP1 [54], [55]. Up-regulation of the PI3K/Akt activity is associated with poor clinical outcome of prostate cancer [33], [34], [50]. c-Myc protein, a well-known proto-oncoprotein, is an important transcriptional regulator of the androgenic response and cell proliferation in prostate and other cancers [25], [56]. Expression of both c-Myc mRNA and protein increase during the progression of prostate cancer [57], [58]. Decrease in c-Myc protein abundance results in decreased prostate cancer cell proliferation and tumor growth [23], [25]–[27], [59]. Forced expression of FASN promoted proliferation while siRNA knockdown of FASN induced apoptosis in prostate cancer cells [60]. Because triol treatment suppressed Akt1, phospho-Akt Ser473, phospho-Akt Thr308, PDK1, c-Myc, and FASN, reduction of Akt signaling, c-Myc, and FASN may all contribute to growth inhibition caused by triol. The only difference in results that we observed between MWAs and conventional Western blots was in the detection of phospho-p44/42 MAPK Thr202/Tyr204, possibly due to the low abundance of the Erk1/2 proteins in these prostate cancer cells and the relatively weak signal of the reporter antibody for this protein. Western blotting also indicated the induction of p27^Kip1^ under triol treatment. The p27^Kip1^ is a cell cycle inhibitory proteins and accumulation of p27^Kip1^ can result in cell cycle arrest and reduced cell proliferation of prostate cancer cells [23], [26], [61]. Skp2 is a member of the F-box protein family that is responsible for ubiquitination and down-regulation of p27^Kip1^ and other proteins [62], [63]. Our observation that triol treatment resulted in decreased Skp2, increased p27^Kip1^, and G1 cell cycle arrest was consistent with the known functions of Skp2 and p27^Kip1^ (Fig. 4, Fig.7, Fig. 8). Overexpression of Skp2 partially rescued the growth inhibition induced by triol (Fig. 9), indicating that other pathways or mechanisms, such as LXR signaling (Fig. 11) or cytoskeletal signaling (Fig. 12), were probably involved in the growth inhibition caused by triol in prostate cancer cells.

Our study suggested that triol treatment causes both G1 cell cycle arrest and apoptosis (Fig. 4, Fig. 5) in CDXR-3, DU-145, and PC-3 *in vitro*. Administration via gavage of 20 mg/kg/day triol five times a week retarded the growth of PC-3 xenografts (Fig. 3). The actual concentration of triol in serum will require further examination. However, the fact that Ki-67 was not significantly reduced in PC-3 tumors suggested the possibility that bioavailability of triol might be poor via oral administration. Local injection of triol might be a more efficient way to administer triol in future studies.

Triol was found to exert oxidative stress in rat hepatocytes as well as to increase levels of superoxide dismutase, catalase, and glutathione peroxidase activity [64]. Components of the cytoskeletal network, such as the actin microfilament meshwork or intermediate filaments, are known targets for oxidative and thiol-depleting agents that induce cell injury. Treatments with 20 µM triol, 7-ketocholesterol, or 25-hydroxycholesterol for 6 hrs caused progressive disruption of actin microfilaments, loss of vinculin, redistribution of vimentin, induction of cell detachment, and stimulation of apoptosis in 73/73 endothelial cells [35], [36]. In our study, confocal laser microscopy revealed a modification of the β-actin and α-tubulin microfilaments, especially redistribution at the periphery of the cell following 48 hrs of treatment with 10 µM triol in CDXR-3 and DU-145 cells (Fig. 8). Alterations of actin and tubulin distribution have been reported to cause cell cycle arrest in human cancer cells [65], [66]. Abnormal distribution of cytoskeletal components caused by triol may contribute to the induction of cell cycle arrest in human prostate cancer cells.

Different signaling proteins in different prostate cancer cells responded differently to triol treatment (Fig. 6–8, 15). Several proteins in DU-145 responded oppositely to 20 µM triol treatment compared to PC-3 and CDXR-3 cells (Fig. 7). This observation might be due to the different characteristics of PC-3, DU-145, and CDXR-3 cells. Although triol treatment suppressed cell proliferation, migration, invasion, and tumor growth in all three of these different prostate cancer cells, it may target different signaling proteins involved in the same pathways (such as Akt signaling) or similar functions (such as regulation of cell cycle, EMT, and cell structure) to produce similar results in different cell lines.

### Conclusions

Our study provided systems-level insight into the molecular mechanism of triol action that resulted in suppression of proliferation, migration, and invasion of prostate cancer cells. Our results suggested that triol might be a potential therapeutic agent for treatment of advanced metastatic prostate cancer. In addition, our study highlights the power of the MWA approach for rapidly examining the systems level impact of small molecules on protein signaling networks and relating these changes to cell phenotypes.

## Supporting Information

Figure S1 **Effect of triol on the growth of DU-145 xenografts in nude mice.** Male Balb/c nu/nu mice, 6–8 weeks of age, were injected subcutaneously in both flanks with 1×10^6^ DU-145 cells suspended in 0.5 ml of Matrigel (BD Bioscience, Franklin Lakes, NJ, USA). Tumors were measured daily using calipers and volume was calculated using the formula volume = length×width×height×0.52. Tumor volume was allowed to increase to larger than 100 mm^3^ before treatment started. Both control and treatment groups had 3 mice carrying 5 tumors. Mice were given intraperitoneal (i.p.) injection of vehicle (5% DMSO and 1% Tween 20 in water) or 1 mg triol in vehicle daily for 14 days. Tumor volumes were shown as mean ± Standard Error.
(TIF)

Figure S2 **Arrangement of samples on blot of MWA is shown.** For left to right, there were two loading of protein markers, CDXR-3 (control), CDXR-3 (20 µM triol), DU-145 (control), DU-145 (20 µM triol), PC-3 (control), and PC-3 (20 µM triol). The right half blot (well A7-H12) was the technical duplicate of the left half blot (well A1-H6).
(TIF)

Table S1 **EC_50_ of triol to suppress viability and proliferation of prostate cancer cells.** LNCaP CDXR-3, DU-145, and PC-3 cells treated with triol for 48 hrs or 96 hrs were assayed with MTT assay or Hoechst dye-based proliferation assay to determine the EC_50_ of triol to cause growth inhibition.
(TIF)

Table S2 **List of antibodies used in the MWA study.** Information of antibodies and location of antibodies on the antibody plate for MWA blot incubation is shown. The α-tubulin, β-actin, and GAPDH proteins were detected as loading controls.
(TIF)

Table S3 **Relative expression level of signaling proteins in prostate cancer cells assayed with MWA.** Relative abundance of signaling proteins in CDXR-3, DU-145, and PC-3 cells treated with 0 or 20 µM triol for 48 hrs was determined by MWA and shown as list. N/A represents signaling being too weak to be detected. Protein abundance was normalized to the average of α-tubulin, GAPDH, and β-actin. Proteins in different cell lines were normalized to the control condition (no triol treatment) of that cell line.
(TIF)

## References

[1] Hellerstedt BA, Pienta KJ (2002) The current state of hormonal therapy for prostate cancer. CA Cancer J Clin 52: 154–179. 12018929 10.3322/canjclin.52.3.154

[2] Chuu CP, Kokontis JM, Hiipakka RA, Fukuchi J, Lin HP, et al (2011) Androgens as therapy for androgen receptor-positive castration-resistant prostate cancer. J Biomed Sci 18: 63. 21859492 10.1186/1423-0127-18-63 PMC3170584

[3] Gilligan T, Kantoff PW (2002) Chemotherapy for prostate cancer. Urology 60: 94–100; discussion 100. 10.1016/s0090-4295(02)01583-2 12231060

[4] Jusakul A, Yongvanit P, Loilome W, Namwat N, Kuver R (2011) Mechanisms of oxysterol-induced carcinogenesis. Lipids Health Dis 10: 44. 21388551 10.1186/1476-511X-10-44 PMC3061933

[5] Dufour J, Viennois E, De Boussac H, Baron S, Lobaccaro JM (2012) Oxysterol receptors, AKT and prostate cancer. Curr Opin Pharmacol. 10.1016/j.coph.2012.06.012 22819197

[6] Chuu CP (2011) Modulation of liver X receptor signaling as a prevention and therapy for colon cancer. Med Hypotheses 76: 697–699. 21333456 10.1016/j.mehy.2011.01.037

[7] Chuu CP, Hiipakka RA, Kokontis JM, Fukuchi J, Chen RY, et al (2006) Inhibition of tumor growth and progression of LNCaP prostate cancer cells in athymic mice by androgen and liver X receptor agonist. Cancer Res 66: 6482–6486. 16818617 10.1158/0008-5472.CAN-06-0632

[8] Chuu CP, Chen RY, Hiipakka RA, Kokontis JM, Warner KV, et al (2007) The liver X receptor agonist T0901317 acts as androgen receptor antagonist in human prostate cancer cells. Biochem Biophys Res Commun 357: 341–346. 17416342 10.1016/j.bbrc.2007.03.116 PMC2693411

[9] Chuu CP, Kokontis JM, Hiipakka RA, Liao S (2007) Modulation of liver X receptor signaling as novel therapy for prostate cancer. J Biomed Sci 14: 543–553. 17372849 10.1007/s11373-007-9160-8

[10] Chuu CP, Lin HP (2010) Antiproliferative effect of LXR agonists T0901317 and 22(R)-hydroxycholesterol on multiple human cancer cell lines. Anticancer Res 30: 3643–3648. 20944148

[11] Fukuchi J, Kokontis JM, Hiipakka RA, Chuu CP, Liao S (2004) Antiproliferative effect of liver X receptor agonists on LNCaP human prostate cancer cells. Cancer Res 64: 7686–7689. 15520170 10.1158/0008-5472.CAN-04-2332

[12] Ayala-Torres S, Zhou F, Thompson EB (1999) Apoptosis induced by oxysterol in CEM cells is associated with negative regulation of c-myc. Exp Cell Res 246: 193–202. 9882528 10.1006/excr.1998.4308

[13] Biasi F, Chiarpotto E, Sottero B, Maina M, Mascia C, et al (2013) Evidence of cell damage induced by major components of a diet-compatible mixture of oxysterols in human colon cancer CaCo-2 cell line. Biochimie 95: 632–640. 23092829 10.1016/j.biochi.2012.10.011

[14] Kang KA, Chae S, Lee KH, Park MT, Lee SJ, et al (2005) Cytotoxic effect of 7beta-hydroxycholesterol on human NCI-H460 lung cancer cells. Biol Pharm Bull 28: 1377–1380. 16079477 10.1248/bpb.28.1377

[15] Bosisio E, Galli G, Nicosia S, Galli Kienle M (1976) Catabolism of cholesterol by bovine adrenal-cortex enzymes: in vitro formation of oxygenated sterols and side-chain cleavage products. Eur J Biochem 63: 491–497. 1261558 10.1111/j.1432-1033.1976.tb10252.x

[16] Watabe T, Sawahata T (1979) Biotransformation of cholesterol to cholestane-3beta,5alpha,6beta-triol via cholesterol alpha-epoxide (5alpha,6alpha-epoxycholestan-3beta-ol) in bovine adrenal cortex. J Biol Chem 254: 3854–3860. 35533

[17] Chevrier N, Mertins P, Artyomov MN, Shalek AK, Iannacone M, et al (2011) Systematic Discovery of TLR Signaling Components Delineates Viral-Sensing Circuits. Cell 147: 853–867. 22078882 10.1016/j.cell.2011.10.022 PMC3809888

[18] Ciaccio MF, Wagner JP, Chuu CP, Lauffenburger DA, Jones RB (2010) Systems analysis of EGF receptor signaling dynamics with microwestern arrays. Nat Methods 7: 148–155. 20101245 10.1038/nmeth.1418 PMC2881471

[19] Hause RJ, Kim HD, Leung KK, Jones RB (2011) Targeted protein-omic methods are bridging the gap between proteomic and hypothesis-driven protein analysis approaches. Expert Rev Proteomics 8: 565–575. 21999828 10.1586/epr.11.49 PMC3269123

[20] Liu J, Kuo WL, Seiwert TY, Lingen M, Ciaccio MF, et al (2011) Effect of complementary pathway blockade on efficacy of combination enzastaurin and rapamycin. Head Neck 33: 1774–1782. 21438065 10.1002/hed.21701

[21] Chuu CP, Chen RY, Kokontis JM, Hiipakka RA, Liao S (2009) Suppression of androgen receptor signaling and prostate specific antigen expression by (−)-epigallocatechin-3-gallate in different progression stages of LNCaP prostate cancer cells. Cancer Lett 275: 86–92. 18977589 10.1016/j.canlet.2008.10.001 PMC2980538

[22] Chuu CP, Hiipakka RA, Fukuchi J, Kokontis JM, Liao S (2005) Androgen causes growth suppression and reversion of androgen-independent prostate cancer xenografts to an androgen-stimulated phenotype in athymic mice. Cancer Res 65: 2082–2084. 15781616 10.1158/0008-5472.CAN-04-3992

[23] Chuu CP, Kokontis JM, Hiipakka RA, Fukuchi J, Lin HP, et al (2011) Androgen suppresses proliferation of castration-resistant LNCaP 104-R2 prostate cancer cells through androgen receptor, Skp2, and c-Myc. Cancer Sci 102: 2022–2028. 21781227 10.1111/j.1349-7006.2011.02043.x PMC3200457

[24] Chuu CP, Lin HP, Ciaccio MF, Kokontis JM, Hause RJ Jr, et al (2012) Caffeic acid phenethyl ester suppresses the proliferation of human prostate cancer cells through inhibition of p70S6K and Akt signaling networks. Cancer Prev Res (Phila) 5: 788–797. 22562408 10.1158/1940-6207.CAPR-12-0004-T PMC4962698

[25] Kokontis J, Takakura K, Hay N, Liao S (1994) Increased androgen receptor activity and altered c-myc expression in prostate cancer cells after long-term androgen deprivation. Cancer Res 54: 1566–1573. 7511045

[26] Kokontis JM, Hay N, Liao S (1998) Progression of LNCaP prostate tumor cells during androgen deprivation: hormone-independent growth, repression of proliferation by androgen, and role for p27Kip1 in androgen-induced cell cycle arrest. Mol Endocrinol 12: 941–953. 9658399 10.1210/mend.12.7.0136

[27] Kokontis JM, Hsu S, Chuu CP, Dang M, Fukuchi J, et al (2005) Role of androgen receptor in the progression of human prostate tumor cells to androgen independence and insensitivity. Prostate 65: 287–298. 16015608 10.1002/pros.20285

[28] Lin HP, Jiang SS, Chuu CP (2012) Caffeic Acid Phenethyl Ester Causes p21 Induction, Akt Signaling Reduction, and Growth Inhibition in PC-3 Human Prostate Cancer Cells. PLoS One 7: e31286. 22347457 10.1371/journal.pone.0031286 PMC3274546

[29] Lin HP, Kuo LK, Chuu CP (2011) Combined Treatment of Curcumin and Small Molecule Inhibitors Suppresses Proliferation of A549 and H1299 Human Non-Small-Cell Lung Cancer Cells. Phytother Res 26: 122–126. 21567511 10.1002/ptr.3523

[30] Stone KR, Mickey DD, Wunderli H, Mickey GH, Paulson DF (1978) Isolation of a human prostate carcinoma cell line (DU 145). Int J Cancer 21: 274–281. 631930 10.1002/ijc.2910210305

[31] Kaighn ME, Narayan KS, Ohnuki Y, Lechner JF, Jones LW (1979) Establishment and characterization of a human prostatic carcinoma cell line (PC-3). Invest Urol 17: 16–23. 447482

[32] Sarker D, Reid AH, Yap TA, de Bono JS (2009) Targeting the PI3K/AKT pathway for the treatment of prostate cancer. Clin Cancer Res 15: 4799–4805. 19638457 10.1158/1078-0432.CCR-08-0125

[33] Lin HP, Lin CY, Liu CC, Su LC, Huo C, et al (2013) Caffeic Acid phenethyl ester as a potential treatment for advanced prostate cancer targeting akt signaling. International journal of molecular sciences 14: 5264–5283. 23466879 10.3390/ijms14035264 PMC3634405

[34] Kreisberg JI, Malik SN, Prihoda TJ, Bedolla RG, Troyer DA, et al (2004) Phosphorylation of Akt (Ser473) is an excellent predictor of poor clinical outcome in prostate cancer. Cancer Res 64: 5232–5236. 15289328 10.1158/0008-5472.CAN-04-0272

[35] Palladini G, Finardi G, Bellomo G (1996) Disruption of actin microfilament organization by cholesterol oxides in 73/73 endothelial cells. Exp Cell Res 223: 72–82. 8635497 10.1006/excr.1996.0059

[36] Palladini G, Finardi G, Bellomo G (1996) Modifications of vimentin filament architecture and vimentin-nuclear interactions by cholesterol oxides in 73/73 endothelial cells. Exp Cell Res 223: 83–90. 8635498 10.1006/excr.1996.0060

[37] Moriel P, Sevanian A, Ajzen S, Zanella MT, Plavnik FL, et al (2002) Nitric oxide, cholesterol oxides and endothelium-dependent vasodilation in plasma of patients with essential hypertension. Braz J Med Biol Res 35: 1301–1309. 12426629 10.1590/s0100-879x2002001100007

[38] Ibrahim T, Flamini E, Mercatali L, Sacanna E, Serra P, et al (2010) Pathogenesis of osteoblastic bone metastases from prostate cancer. Cancer 116: 1406–1418. 20108337 10.1002/cncr.24896

[39] Keller ET, Zhang J, Cooper CR, Smith PC, McCauley LK, et al (2001) Prostate carcinoma skeletal metastases: cross-talk between tumor and bone. Cancer Metastasis Rev 20: 333–349. 12085970 10.1023/a:1015599831232

[40] Bubendorf L, Schopfer A, Wagner U, Sauter G, Moch H, et al (2000) Metastatic patterns of prostate cancer: an autopsy study of 1,589 patients. Hum Pathol 31: 578–583. 10836297 10.1053/hp.2000.6698

[41] Graham TR, Zhau HE, Odero-Marah VA, Osunkoya AO, Kimbro KS, et al (2008) Insulin-like growth factor-I-dependent up-regulation of ZEB1 drives epithelial-to-mesenchymal transition in human prostate cancer cells. Cancer Res 68: 2479–2488. 18381457 10.1158/0008-5472.CAN-07-2559

[42] Acevedo VD, Gangula RD, Freeman KW, Li R, Zhang Y, et al (2007) Inducible FGFR-1 activation leads to irreversible prostate adenocarcinoma and an epithelial-to-mesenchymal transition. Cancer Cell 12: 559–571. 18068632 10.1016/j.ccr.2007.11.004

[43] Xu J, Wang R, Xie ZH, Odero-Marah V, Pathak S, et al (2006) Prostate cancer metastasis: role of the host microenvironment in promoting epithelial to mesenchymal transition and increased bone and adrenal gland metastasis. Prostate 66: 1664–1673. 16902972 10.1002/pros.20488

[44] Ao M, Williams K, Bhowmick NA, Hayward SW (2006) Transforming growth factor-beta promotes invasion in tumorigenic but not in nontumorigenic human prostatic epithelial cells. Cancer Res 66: 8007–8016. 16912176 10.1158/0008-5472.CAN-05-4451 PMC4067141

[45] Beach S, Tang H, Park S, Dhillon AS, Keller ET, et al (2008) Snail is a repressor of RKIP transcription in metastatic prostate cancer cells. Oncogene 27: 2243–2248. 17952120 10.1038/sj.onc.1210860 PMC2933472

[46] Sakamoto S, McCann RO, Dhir R, Kyprianou N (2010) Talin1 promotes tumor invasion and metastasis via focal adhesion signaling and anoikis resistance. Cancer Research 70: 1885–1895. 20160039 10.1158/0008-5472.CAN-09-2833 PMC2836205

[47] Zheng DQ, Woodard AS, Fornaro M, Tallini G, Languino LR (1999) Prostatic carcinoma cell migration via alpha(v)beta3 integrin is modulated by a focal adhesion kinase pathway. Cancer Research 59: 1655–1664. 10197643

[48] Senapati S, Rachagani S, Chaudhary K, Johansson SL, Singh RK, et al (2010) Overexpression of macrophage inhibitory cytokine-1 induces metastasis of human prostate cancer cells through the FAK-RhoA signaling pathway. Oncogene 29: 1293–1302. 19946339 10.1038/onc.2009.420 PMC2896817

[49] Franzen CA, Amargo E, Todorovic V, Desai BV, Huda S, et al (2009) The chemopreventive bioflavonoid apigenin inhibits prostate cancer cell motility through the focal adhesion kinase/Src signaling mechanism. Cancer prevention research 2: 830–841. 19737984 10.1158/1940-6207.CAPR-09-0066

[50] Liu CC, Hsu JM, Kuo LK, Chuu CP (2013) Caffeic acid phenethyl ester as an adjuvant therapy for advanced prostate cancer. Medical hypotheses 80: 617–619. 23462369 10.1016/j.mehy.2013.02.003

[51] Coffer PJ, Jin J, Woodgett JR (1998) Protein kinase B (c-Akt): a multifunctional mediator of phosphatidylinositol 3-kinase activation. Biochem J 335 (Pt 1): 1–13. 10.1042/bj3350001 PMC1219745 9742206

[52] Gonzalez E, McGraw TE (2009) The Akt kinases: isoform specificity in metabolism and cancer. Cell Cycle 8: 2502–2508. 19597332 10.4161/cc.8.16.9335 PMC2997486

[53] Alessi DR, James SR, Downes CP, Holmes AB, Gaffney PR, et al (1997) Characterization of a 3-phosphoinositide-dependent protein kinase which phosphorylates and activates protein kinase Balpha. Curr Biol 7: 261–269. 9094314 10.1016/s0960-9822(06)00122-9

[54] Sarbassov DD, Guertin DA, Ali SM, Sabatini DM (2005) Phosphorylation and regulation of Akt/PKB by the rictor-mTOR complex. Science 307: 1098–1101. 15718470 10.1126/science.1106148

[55] Jacinto E, Facchinetti V, Liu D, Soto N, Wei S, et al (2006) SIN1/MIP1 maintains rictor-mTOR complex integrity and regulates Akt phosphorylation and substrate specificity. Cell 127: 125–137. 16962653 10.1016/j.cell.2006.08.033

[56] Vellaichamy A, Dezso Z, JeBailey L, Chinnaiyan AM, Sreekumar A, et al (2010) "Topological significance" analysis of gene expression and proteomic profiles from prostate cancer cells reveals key mechanisms of androgen response. PLoS One 5: e10936. 20532174 10.1371/journal.pone.0010936 PMC2880599

[57] Edwards J, Krishna NS, Witton CJ, Bartlett JM (2003) Gene amplifications associated with the development of hormone-resistant prostate cancer. Clin Cancer Res 9: 5271–5281. 14614009

[58] Grad JM, Dai JL, Wu S, Burnstein KL (1999) Multiple androgen response elements and a Myc consensus site in the androgen receptor (AR) coding region are involved in androgen-mediated up-regulation of AR messenger RNA. Mol Endocrinol 13: 1896–1911. 10551783 10.1210/mend.13.11.0369

[59] Umekita Y, Hiipakka RA, Kokontis JM, Liao S (1996) Human prostate tumor growth in athymic mice: inhibition by androgens and stimulation by finasteride. Proc Natl Acad Sci U S A 93: 11802–11807. 8876218 10.1073/pnas.93.21.11802 PMC38139

[60] Migita T, Ruiz S, Fornari A, Fiorentino M, Priolo C, et al (2009) Fatty acid synthase: a metabolic enzyme and candidate oncogene in prostate cancer. J Natl Cancer Inst 101: 519–532. 19318631 10.1093/jnci/djp030 PMC2664091

[61] Elledge SJ, Harper JW (1994) Cdk inhibitors: on the threshold of checkpoints and development. Curr Opin Cell Biol 6: 847–852. 7880532 10.1016/0955-0674(94)90055-8

[62] Carrano AC, Eytan E, Hershko A, Pagano M (1999) SKP2 is required for ubiquitin-mediated degradation of the CDK inhibitor p27. Nat Cell Biol 1: 193–199. 10559916 10.1038/12013

[63] Tsvetkov LM, Yeh KH, Lee SJ, Sun H, Zhang H (1999) p27(Kip1) ubiquitination and degradation is regulated by the SCF(Skp2) complex through phosphorylated Thr187 in p27. Curr Biol 9: 661–664. 10375532 10.1016/s0960-9822(99)80290-5

[64] Cantwell H, Devery R (1998) The response of the antioxidant defense system in rat hepatocytes challenged with oxysterols is modified by Covi-ox. Cell Biol Toxicol 14: 401–409. 9879932 10.1023/a:1007595527176

[65] Panagiotou S, Bakogeorgou E, Papakonstanti E, Hatzoglou A, Wallet F, et al (1999) Opioid agonists modify breast cancer cell proliferation by blocking cells to the G2/M phase of the cycle: involvement of cytoskeletal elements. J Cell Biochem 73: 204–211. 10227383 10.1002/(sici)1097-4644(19990501)73:2<204::aid-jcb6>3.0.co;2-v

[66] Nolasco S, Bellido J, Goncalves J, Zabala JC, Soares H (2005) Tubulin cofactor A gene silencing in mammalian cells induces changes in microtubule cytoskeleton, cell cycle arrest and cell death. FEBS letters 579: 3515–3524. 15963512 10.1016/j.febslet.2005.05.022

