# Supplementary figures and images for: Cholestane-3β, 5α, 6β-triol Suppresses Proliferation, Migration, and Invasion of Human Prostate Cancer Cells
Source: PLoS One. 2013 Jun 13;8(6):e65734. doi: 10.1371/journal.pone.0065734 (PMC3681800; doi:10.1371/journal.pone.0065734)

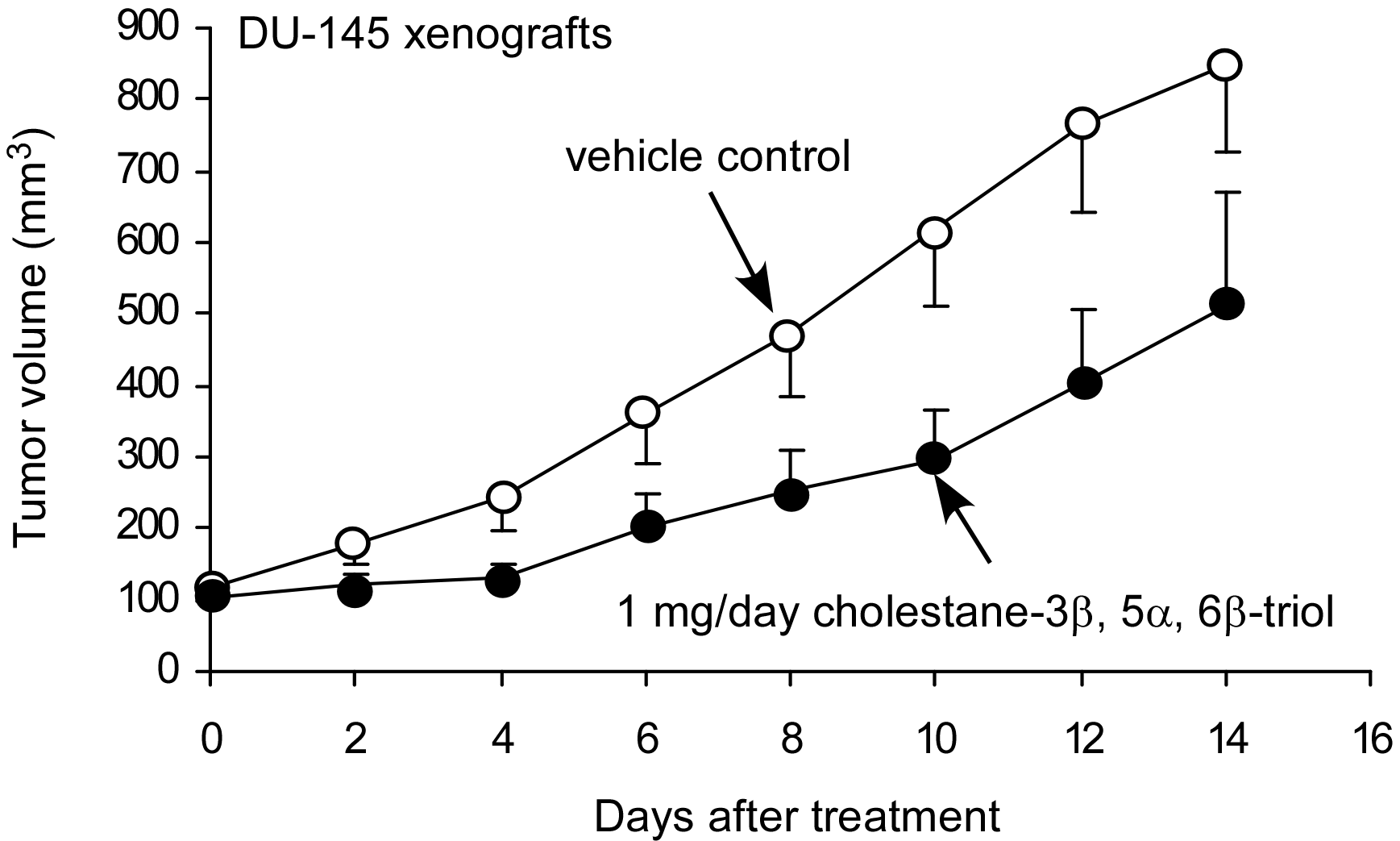

Supplement: Figure S1 — Effect of triol on the growth of DU-145 xenografts in nude mice. Male Balb/c nu/nu mice, 6–8 weeks of age, were injected subcutaneously in both flanks with 1×10^6^ DU-145 cells suspended in 0.5 ml of Matrigel (BD Bioscience, Franklin Lakes, NJ, USA). Tumors were measured daily using calipers and volume was calculated using the formula volume = length×width×height×0.52. Tumor volume was allowed to increase to larger than 100 mm3 before treatment started. Both control and treatment groups had 3 mice carrying 5 tumors. Mice were given intraperitoneal (i.p.) injection of vehicle (5% DMSO and 1% Tween 20 in water) or 1 mg triol in vehicle daily for 14 days. Tumor volumes were shown as mean ± Standard Error. (TIF) [file pone.0065734.s001.tif]

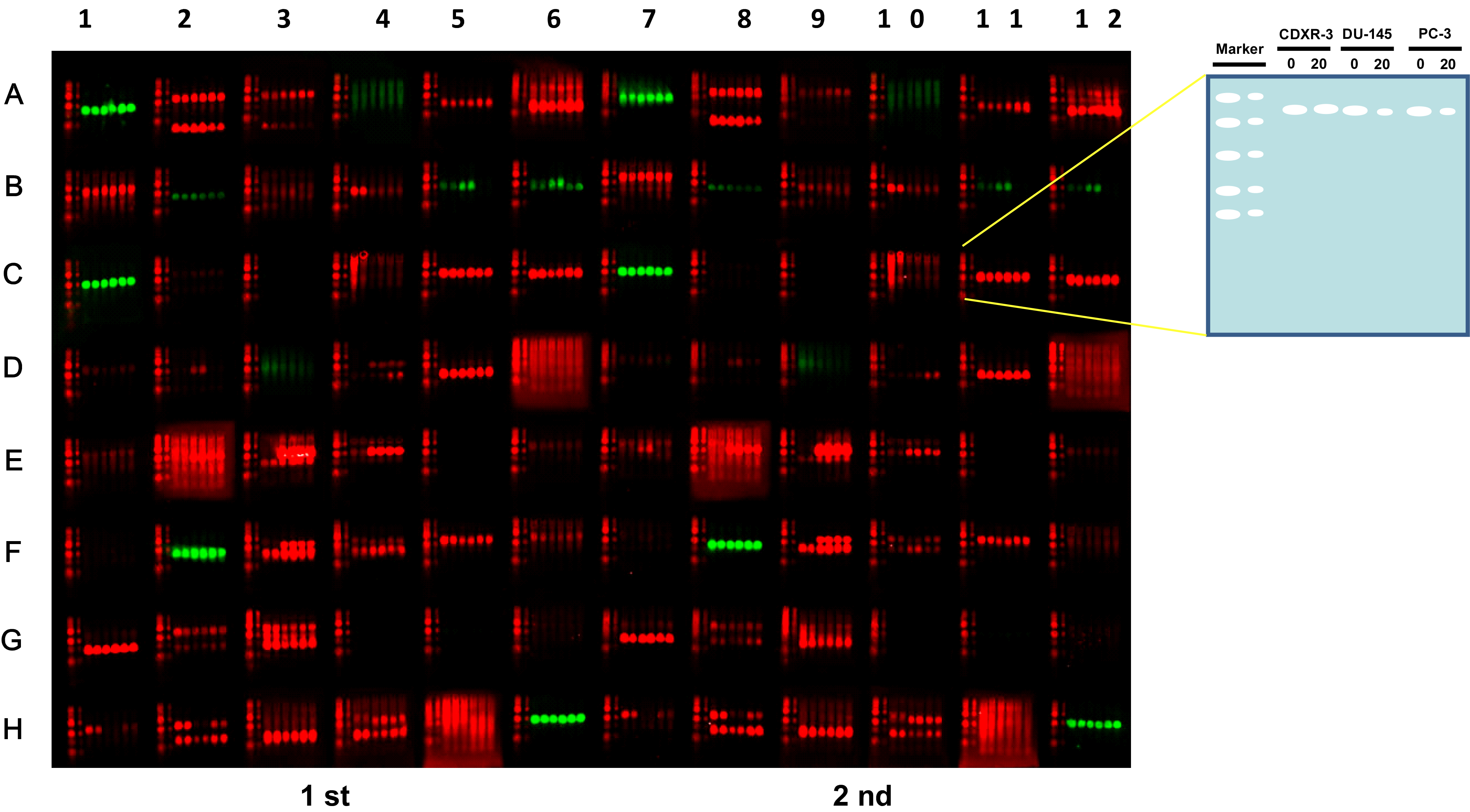

Supplement: Figure S2 — Arrangement of samples on blot of MWA is shown. For left to right, there were two loading of protein markers, CDXR-3 (control), CDXR-3 (20 µM triol), DU-145 (control), DU-145 (20 µM triol), PC-3 (control), and PC-3 (20 µM triol). The right half blot (well A7-H12) was the technical duplicate of the left half blot (well A1-H6). (TIF) [file pone.0065734.s002.tif]

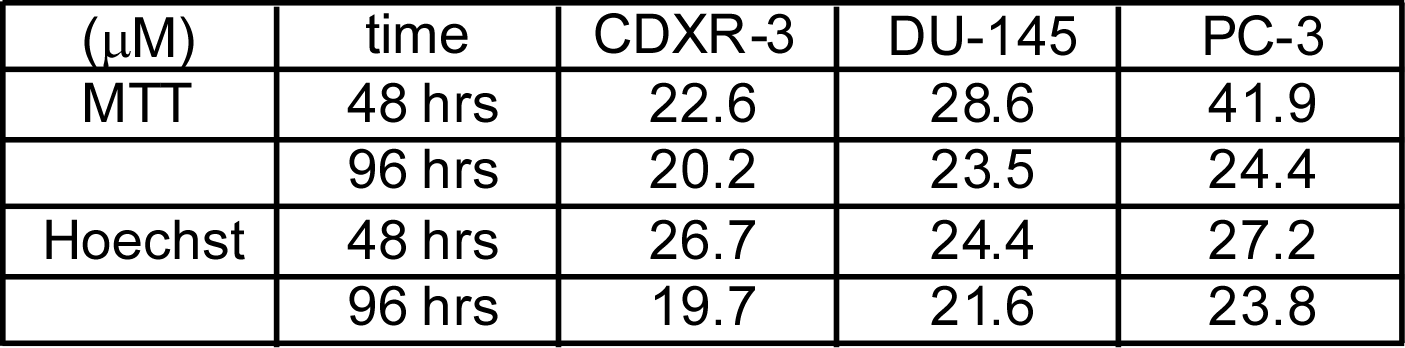

Supplement: Table S1 — EC50 of triol to suppress viability and proliferation of prostate cancer cells. LNCaP CDXR-3, DU-145, and PC-3 cells treated with triol for 48 hrs or 96 hrs were assayed with MTT assay or Hoechst dye-based proliferation assay to determine the EC50 of triol to cause growth inhibition. (TIF) [file pone.0065734.s003.tif]

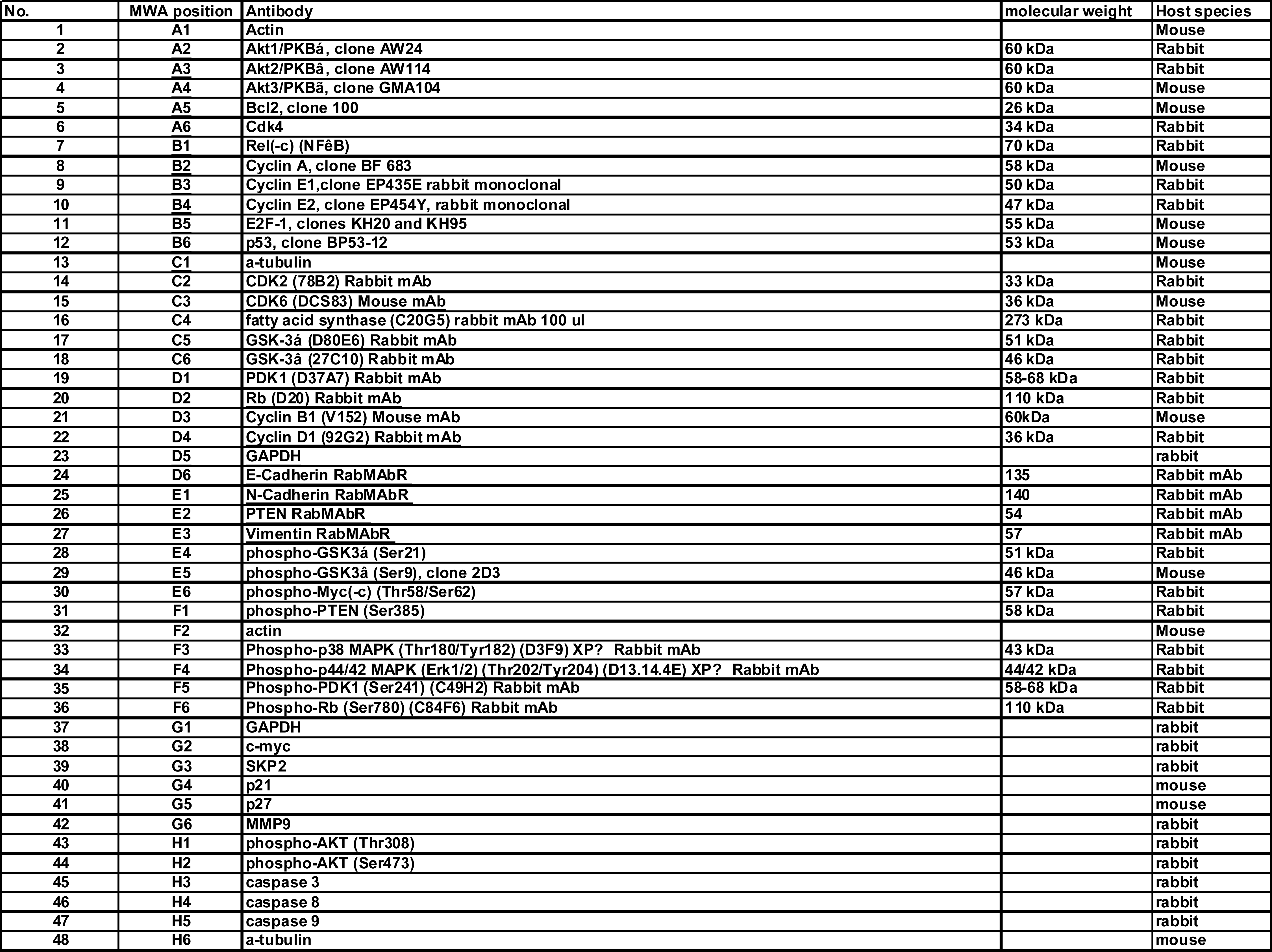

Supplement: Table S2 — List of antibodies used in the MWA study. Information of antibodies and location of antibodies on the antibody plate for MWA blot incubation is shown. The α-tubulin, β-actin, and GAPDH proteins were detected as loading controls. (TIF) [file pone.0065734.s004.tif]

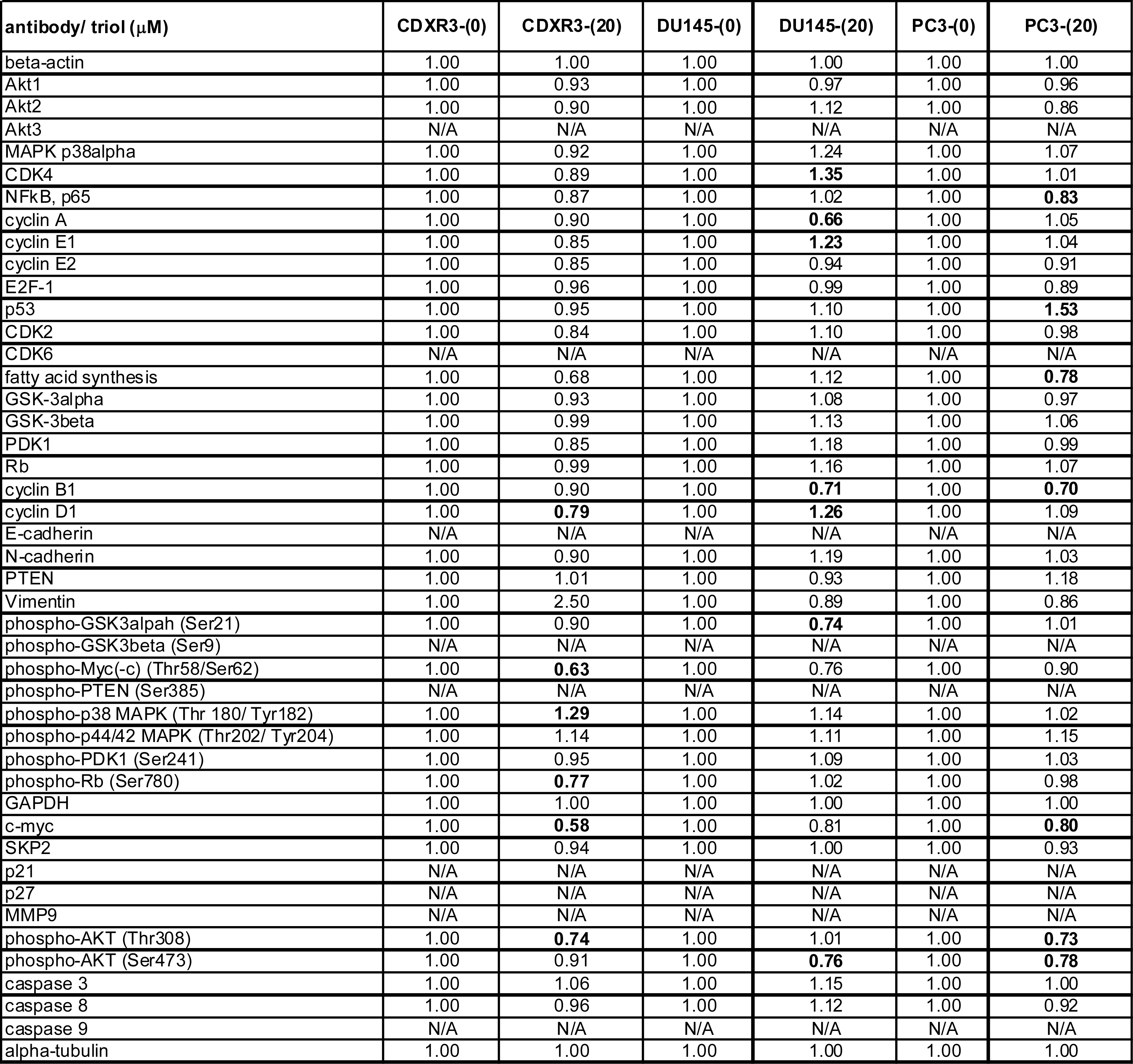

Supplement: Table S3 — Relative expression level of signaling proteins in prostate cancer cells assayed with MWA. Relative abundance of signaling proteins in CDXR-3, DU-145, and PC-3 cells treated with 0 or 20 µM triol for 48 hrs was determined by MWA and shown as list. N/A represents signaling being too weak to be detected. Protein abundance was normalized to the average of α-tubulin, GAPDH, and β-actin. Proteins in different cell lines were normalized to the control condition (no triol treatment) of that cell line. (TIF) [file pone.0065734.s005.tif]
